# Contacts with primary and secondary healthcare before suicide by those under the care of mental health services: case–control, whole-population-based study using person-level linked routine data in Wales, UK during 2000–2015

**DOI:** 10.1192/bjo.2024.23

**Published:** 2024-05-10

**Authors:** Marcos DelPozo-Banos, Cathryn Rodway, Sze Chim Lee, Olivier Y. Rouquette, Saied Ibrahim, Keith Lloyd, Louis Appleby, Navneet Kapur, Ann John

**Affiliations:** Swansea University Medical School, UK; National Confidential Inquiry into Suicide and Safety in Mental Health (NCISH), Centre for Mental Health and Safety, School of Health Sciences, University of Manchester, UK; National Confidential Inquiry into Suicide and Safety in Mental Health (NCISH), Centre for Mental Health and Safety, School of Health Sciences, University of Manchester, UK; NIHR Greater Manchester Patient Safety Research Collaboration, University of Manchester, UK; and Mersey Care NHS Foundation Trust, Prescot, UK

**Keywords:** Suicide, electronic health records, primary care, secondary care, mental health services

## Abstract

**Background:**

People under the care of mental health services are at increased risk of suicide. Existing studies are small in scale and lack comparisons.

**Aims:**

To identify opportunities for suicide prevention and underpinning data enhancement in people with recent contact with mental health services.

**Method:**

This population-based study includes people who died by suicide in the year following a mental health services contact in Wales, 2001–2015 (cases), paired with similar patients who did not die by suicide (controls). We linked the National Confidential Inquiry into Suicide and Safety in Mental Health and the Suicide Information Database – Cymru with primary and secondary healthcare records. We present results of conditional logistic regression.

**Results:**

We matched 1031 cases with 5155 controls. In the year before their death, 98.3% of cases were in contact with healthcare services, and 28.5% presented with self-harm. Cases had more emergency department contacts (odds ratio 2.4, 95% CI 2.1–2.7) and emergency hospital admissions (odds ratio 1.5, 95% CI 1.4–1.7), but fewer primary care contacts (odds ratio 0.7, 95% CI 0.6–0.9) and out-patient appointments (odds ratio 0.2, 95% CI 0.2–0.3) than controls. Odds ratios were larger in females than males for injury and poisoning (odds ratio: 3.3 (95% CI 2.5–4.5) *v.* 2.6 (95% CI 2.1–3.1)).

**Conclusions:**

We may be missing existing opportunities to intervene, particularly in emergency departments and hospital admissions with self-harm presentations and with unattributed self-harm, especially in females. Prevention efforts should focus on strengthening routine care contacts, responding to emergency contacts and better self-harm care. There are benefits to enhancing clinical audit systems with routinely collected data.

People under the care of mental health services are at increased risk of suicide.^1^ This risk is heightened following discharge from psychiatric in-patient care,^2^ and more so in the first days post-discharge, peaking at day 3.^3^ Consequently, the National Confidential Inquiry into Suicide and Safety in Mental Health (NCISH) in the UK recommended a follow-up period within 72 h of discharge,^4^ included on the standard National Health Service (NHS) contract in England.^5^ UK's National Institute for Health and Care Excellence (NICE) guidelines recommend follow-up within 48 h for patients identified as at risk of suicide.^6^ Yet, almost one in four of those who die by suicide in the UK following a contact with a mental health specialist may not receive relevant interventions in this period.^7^

In England, in the year before their deaths by suicide, the majority of patients in current or recent contact with secondary mental health services were also seen in primary care (general practice), some as many as seven times.^8^ Similarly, two in five patients were seen in emergency departments, some as many as three or more times.^9^ However, not all patients presenting to these settings with mental health conditions go on to take their own lives, and identifying those at highest risk is highly challenging for practitioners. Of those seen in primary care in the year before their death, only 3% received a moderate-to-high risk level after assessment by both their general practitioners (GPs) and their mental health team.^8^ Similarly, there is no evidence on the effectiveness of risk assessment tools and scales for screening for suicide risk in these settings, and the latest NICE guidance recommends they should not be used to predict risk of self-harm or suicide, nor to decide levels of care.^10^

Case–control studies point to factors such as previous suicide attempts,^11,13^ history of or current self-harm,^12,14^ non-adherence to treatment^13,14^ and adverse life events^15^ as risk factors for suicide in psychiatric in-patients and those recently discharged. The most reported risk factor is self-discharge.^14,16,17^ However, many of these studies are small in scale, limited to data from a single setting and/or have poor comparison data.

In this study, we linked clinical audit data with routinely collected electronic health records, and explored any benefits for suicide prevention research, including access to population-based controls; improved data completion; and increased variables, history coverage and level of detail. We conducted a case–control study of patients under the care of mental health services who died by suicide in the subsequent 12 months, defining population-based controls in contact with such services and with similar mental health diagnosis; and linking questionnaire data to primary and secondary care person-level, routinely collected data covering the whole of Wales for 15 years (2000–2015). To the best of our knowledge, this is the first study combining all of these characteristics.

## Aims

We aimed to identify opportunities for suicide prevention in people with recent contact with mental health services, and whether there are any related benefits from linking clinical audit systems and routinely collected data.

## Method

### Study design

For all those seen by mental health services in Wales, we compared healthcare contacts and diagnoses of those who died by suicide in the following year (identified in the NCISH data-set, a national audit system, and the SID-Cymru data-set, based on routinely collected electronic records) with those who did not die by suicide. We conducted a case–control study of deaths by suicide in residents of Wales aged ≥10 years between 1 January 2001 and 31 December 2015. Cases included individuals identified through either data source. Wales has a population of 3.1 million, with 33 000 annual deaths, of which around 350 (1%) are suicide.^18^ Suicide is defined as fatal intentional self-harm (ICD-10 codes X60–X84) at age ≥10 years and events of undetermined intent (ICD-10 codes Y10–Y34, Y87 and Y87.2; excluding Y33.9 before 2007) at age ≥15 years, as is conventional practice in UK.^18^

### Data-sets

Suicide deaths were obtained from the NCISH and the Suicide Information Database – Wales (SID-Cymru), both hosted in the Secure Anonymised Information Linkage (SAIL) Databank (www.saildatabank.com), where they were linked with primary and secondary healthcare data (detailed below).

#### NCISH

NCISH collects UK-wide data on a consecutive case series of all people who die by suicide while under the recent care of mental health services. National mortality data from the Office for National Statistics (ONS) provide details on all deaths with a coroner's inquest assigning a suicide or undetermined conclusion. Then, of these people, mental health providers identify who had contact with mental health services in the 12 months before death. The senior professional responsible for the patient's care completes a questionnaire with detailed clinical information. NCISH data showed 1117 suicide deaths in Wales over the study period.

#### SID-Cymru

SID-Cymru is a population-based, anonymous electronic cohort of all Welsh residents who die by suicide.^19,20^ It recorded 4654 deaths by suicide over the study period; the expectation was that these included the 1117 suicide deaths reported in the NCISH data.

#### SAIL Databank

SAIL is a privacy-protecting Trusted Research Environment holding anonymised data from the whole population of Wales.^21^ We used 2000–2015 data linked deterministically or probabilistically with a matching score^22^ ≥0.9 from the Welsh Demographic Service, ONS Mortality Register, Wales Longitudinal General Practice Dataset (covering 78% of the population), Emergency Department Dataset, Patient Episode Dataset for Wales and the Outpatient Database for Wales (full details in Supplementary Table 1 available at https://doi.org/10.1192/bjo.2024.23). Records from the NCISH were linked within SAIL, which also hosts SID-Cymru.

### Case and control definitions

We defined suicide cases as deaths recorded in the NCISH data-set (‘NCISH cases’), as well as those not in the NCISH data-set but in SID-Cymru with a mental health service contact recorded in SAIL in the year before death (‘SID-Cymru-only cases’). For each case, we defined the index date as the date of death or, if present, the start date of the healthcare contact where the patient died (e.g. during a psychiatric admission, following an emergency admission resulting from overdose, etc.).

Cases met ‘data availability inclusion criteria’ if they had age, gender and date of death recorded; had an index date in 2001–2015; and were living in Wales in the year before death and in the year before the index date.

Within SAIL, we matched suicide cases to five live controls who were also in contact with mental health services in the year before the case's death date. Controls were matched by gender and primary mental health diagnosis (defined below). Controls with similar values of the following variables were given precedence during the selection process: week of birth (maximum difference ±5 years), deprivation index, and Welsh residency period and primary care data coverage before the matching case's death date. This ensured similar quality of data coverage between cases and controls, and recency of matching diagnosis.

We conducted a single-setting analysis (primary care, emergency departments, hospitals, out-patients) using all available data, and an analysis across all four settings from 2010, because of variable data availability. We used (a) individuals with an index date in 2001 or later, registered with a general practice providing data to SAIL in the year before their index date, to study primary care variables; (b) individuals with an index date in 2010 or later, to study emergency department visits; (c) all individuals in the study population (index date in 2001 or later), to study hospital admissions; (d) individuals with an index date in 2005 or later, to study out-patient attendances and (e) individuals with an index date in 2010 or later and registered with a general practice providing data to SAIL in the year before their index date, to simultaneously study contacts across all four settings.

### Measures and variables

#### Demographics

We extracted gender and age (10–24, 25–64, ≥65 years) at death from the Welsh Demographic Service data source. Residential records in primary care data were used to extract level of deprivation and rural/urban context at death. We measured area deprivation at the Lower Layer Super Output Area (LSOA) level (approximately 1500 individuals), using the Welsh Index of Multiple Deprivation (WIMD) 2011.^23^ We defined WIMD deprivation levels 1–5 with national WIMD score quintiles as cut-offs (level 1 being the least deprived areas). Rural/urban context categorises each LSOA area into ‘urban’ (i.e. settlement types with a population of 10 000 or more) and ‘rural’ (i.e. the union of categories of ‘town & fringe’ and ‘village, hamlet and isolated dwellings’).^24^

#### Diagnostic variables

We extracted diagnostic variables before death (for controls, before the case's death) for control matching and the comparison of NCISH and SAIL data. A list of diagnoses readily available in NCISH data can be found in Supplementary Table 2. NCISH data highlighted which of these was the ‘primary diagnosis’, used for control matching. We identified the same diagnoses in SAIL by using primary care data (Read codes)^25^ and hospital admission data (ICD-10)^26^ (code lists are available in Supplementary Table 2).^27,39^

#### Mental health service contacts

We identified secondary care/specialist mental health service contacts in the year before death (for controls, year before the case's death) for control matching and the comparison of NCISH and SAIL data. These included secondary care/specialist mental health service contacts recorded in primary care; hospital admissions with an episode with a psychiatric specialty or treatment, a mental health related Healthcare Resource Group code or a mental health primary diagnosis (ICD-10 codes ‘F’); and out-patient attendances with a psychiatric specialty or treatment.

#### Contact with services

We measured contacts for cases and controls in 1 week, 1 month and 1 year before, but not including, the index date. We defined ‘contact’ as a recorded entry in healthcare records. For primary care only, administrative codes and associated diagnoses such as ‘letter from emergency department', were excluded, but we did include telephone and face-to-face contacts with any member of the primary care team.^20^ Hospital admissions were stratified into emergency and elective admissions.

#### Accidents, injury and poisoning, and self-harm contacts

We identified contacts in primary care, emergency department and hospital admissions for accidents, injury and poisoning (undetermined or unspecified intent), and self-harm in the year before, but not including, the index date by using validated ICD-10 and Read codes lists, reviewed and updated by expert clinicians (Supplementary Table 2).^40^

### Statistical methods

Data in SAIL was interrogated with SQL DB2-11 for Windows (IBM; www.ibm.com/analytics/db2) and analysed with R v4.2.2 for Windows (www.r-project.org). We descriptively compared NCISH and SAIL data sources. We measured the overlap in suicide cases found in either NCISH or SID-Cymru. We identified cases with primary care and hospital admissions and out-patient data in the year before their death, to assess how many had a mental health service contact in the year before death recorded in SAIL. We used cases with primary care and hospital admission data in the year before death, to measure how many of NCISH primary diagnoses were also recorded in SAIL, and the relationship between diagnostic information in NCISH and SAIL data.

We compared contacts with healthcare services between suicide cases and controls. Specifically, the proportions of cases and controls presenting to healthcare services at 1 week, 1 month and 1 year before the index date; and with accidents, injury and poisoning, or self-harm in the year before the index date. We summarised results via descriptive statistics: counts, percentages and 95% confidence intervals estimated by Wilson score, with continuity correction.^41^ We present results separately for males and females, and for NCISH cases and SID-Cymru-only cases, in the Supplementary Material.

When studying presentations to healthcare services, we present odds ratios between cases and controls of univariate conditional logistic regressions for each setting, contact and time window (‘single-window regression’). We also present odds ratios of conditional logistic regression models for each healthcare setting, with multi-window categorical independent variables with levels ‘no contact’, ‘contact between 1 day and 1 week before’, ‘contact between 2 weeks and 1 month before’ and ‘contact between 2 months and 1 year before’ (‘multi-window regression’). This had two independent variables in hospital admissions (emergency and planned admissions) and hospital out-patients (attended and cancelled or missed appointments).

For the study of diagnoses, we present odds ratios of univariate conditional logistic regression for each diagnosis. We also present odds ratios of a multivariate conditional logistic regression per healthcare setting, including independent variables ‘accidents’, ‘injury and poisoning’ and ‘self-harm’.

Finally, we noted the setting of the last contact in the year before the index date and its recorded diagnoses.^20^

### Ethics statement

The authors assert that all procedures contributing to this work comply with the ethical standards of the relevant national and institutional committees. NCISH data were provided by the Healthcare Quality Improvement Partnership from the Mental Health Clinical Outcome Review Programme, as delivered by the NCISH: HQIP200. NCISH received approval from the National Research Ethics Committee North West (Greater Manchester South, UK; approval number 15/NW/0184) and the Health Research Authority Confidentiality Advisory Group (approval number PIAG 4-08(d)/2003), to link NCISH data with the SID-Cymru data. Ethical approval for this research was granted by SAIL's Information Governance Review Panel (approval number 0788). Results presented here were reviewed independently to ensure they comply with SAIL's information governance policies.

## Results

### Study population

**Fig. 1:**
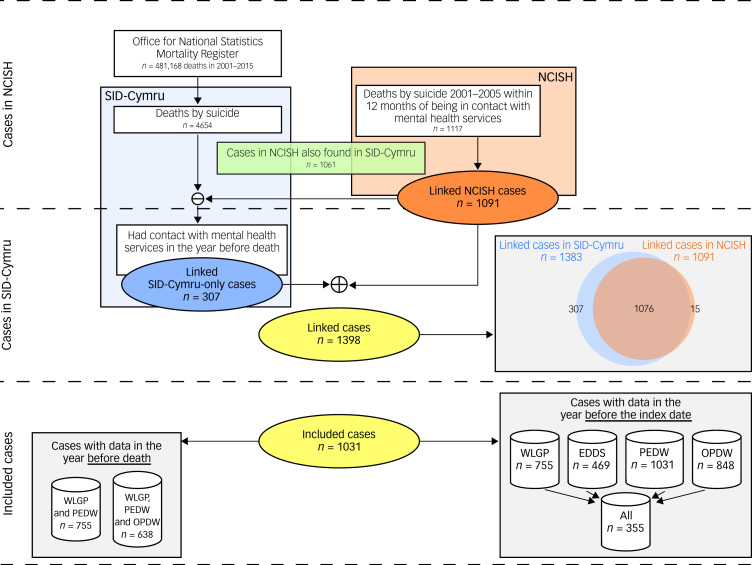
Flow diagram of cases. A more detailed version of this diagram can be seen in Supplementary Fig. 1. NCISH, National Confidential Inquiry into Suicide and Safety in Mental Health; SID-Cymru, Suicide Information Database – Wales; WLGP, Welsh Longitudinal General Practice Dataset; EDDS, Emergency Department Dataset; PEDW, Patient Episode Dataset for Wales; OPDW, Outpatient Database for Wales.

Of the 1117 suicide cases in NCISH, we successfully linked 99.7% (*n* = 1091) to SAIL data. An additional 307 suicide cases in SID-Cymru (‘SID-Cymru only’) had a mental health service contact in the year before death recorded in SAIL. Thus, we identified a total of 1398 suicide cases with a mental health service contact in the year before their death. Of these, 1031 (814 in NCISH and 217 in SID-Cymru-only) met the data availability inclusion criteria, representing the final study cohort (Fig. 1 and Supplementary Fig. 1). Table 1 shows the distribution of demographic variables for these cases and a breakdown for cases found in NCISH or in SID-Cymru-only, with a comparison between cases and controls presented in Supplementary Table 3. The identified index date was earlier than the date of death in 9.9% (*n* = 101; 82 in NCISH and 19 in SID-Cymru-only) of cases. Mental health service contacts recorded in SAIL for cases who had primary care, hospital admission and hospital out-patient data coverage in the year before their death (*n* = 638) can be seen in Table 1. Compared with cases ascertained through NCISH, a higher proportion of SID-Cymru-only cases had mental health service contacts recorded in primary care data, and a lower proportion recorded in hospital admission and out-patient data.

**Table 1.** Demographics of all cases in the study population and a breakdown for cases in NCISH or in SID-Cymru-only, and mental health service contacts in the year before death for those with primary care, hospital admission and hospital out-patient data in this period for the largest subcohort with the minimum required data sources

	All cases, n; % [95% CI]	Cases in NCISH, n; % [95% CI]	Cases in SID-Cymru-only, n; % [95% CI]
Full study population			
Total	1031 (100%)	814 (100%)	217 (100%)
Males/Females			
Females	328; 31.8% [29.0, 34.7]	262; 32.2% [29.1%, 35.5%]	66; 30.4% [24.7%, 36.8%]
Males	703; 68.19% [65.3, 71.0]	552; 67.8% [64.5%, 70.9%]	151; 69.6% [63.2%, 75.3%]
Age groups			
[10, 24] years old	69; 6.7% [5.3, 8.4]	52; 6.4% [4.9%, 8.3%]	17; 7.8% [5.0%, 12.2%]
[25, 64] years old	807; 78.3% [75.7, 80.7]	645; 79.2% [76.3%, 81.9%]	162; 74.7% [68.5%, 80.0%]
64+ years old	155; 15.0% [13.0, 17.3]	117; 14.4% [12.1%, 17.0%]	38; 17.5% [13.0%, 23.1%]
Deprivation index			
Least deprived - 1	158; 15.3% [13.3, 17.7]	125; 15.7% [13.0%, 18.0%]	33; 15.2% [11.0%, 20.6%]
2	170; 16.5% [14.3, 18.9]	136; 16.7% [14.3%, 19.4%]	34; 15.7% [11.4%, 21.1%]
3	205; 19.9% [17.6, 22.4]	154; 18.9% [16.4%, 21.8%]	51; 23.5% [18.4%, 29.6%]
4	246; 23.9% [21.4, 26.6]	195; 24.0% [21.2%, 27.0%]	51; 23.5% [18.4%, 29.6%]
Most deprived - 5	252; 24.4% [21.9, 27.2]	204; 25.1% [22.2%, 28.2%]	48; 22.1% [17.1%, 28.1%]
Missing value	<5; 0.5% [0.2, 1.1]	<5; 0.6% [0.3%, 1.4%]	<5; 2.3% [1.0%, 5.3%]
Area morphology			
Urban	719; 69.7% [66.9, 72.5]	561; 68.9% [65.6%, 72.0%]	158; 72.8% [66.5%, 78.3%]
Rural	182; 17.7% [15.4, 20.1]	149; 18.3% [15.8%, 21.1%]	33; 15.2% [11.0%, 20.6%]
Missing value	<5; 0.5% [0.2, 1.1]	<5; 0.6% [0.3%, 1.4%]	<5; 2.3% [1.0%, 5.3%]
Study population with WLGP, PEDW and OPDW data in the year before death			
Total	638 (100%)	485 (100%)	153 (100%)
Mental health service contacts in primary care data			
Recorded contact	245; 38.4% [37.7, 42.2]	169; 34.8% [30.7%, 39.2%]	76; 49.7% [41.9%, 57.5%]
Mental health service contacts in hospital admissions data			
Specialty	238; 38.4% [34.7, 42.2]	203; 41.7% [37.5%, 46.3%]	35; 22.9% [16.9%, 30.1%]
Health Resource Group	89; 13.9% [11.5, 16.9]	69; 14.2% [11.4%, 17.6%]	20; 13.1% [8.6%, 19.3%]
Main diagnosis	207; 32.4% [28.9, 36.2]	169; 34.8% [30.7%, 39.2%]	38; 24.8% [18.7%, 32.2%]
Any of the above	265; 41.5% [37.8, 45.4]	212; 43.7% [39.4%, 48.2%]	53; 34.6% [27.6%, 42.5%]
Mental health service contacts in hospital outpatients data			
Specialty	305; 47.8% [44.0, 51.7]	238; 49.1% [44.6%, 53.5%]	67; 43.8% [36.2%, 51.7%]
Mental health service contacts recorded			
Only in primary care	94; 14.7% [12.2, 17.7]	45; 9.3% [7.0%, 12.2%]	49; 32.0% [25.2%, 39.8%]
Only in hospital admissions	105; 16.5% [13.8, 19.5]	74; 15.3% [12.3%, 18.7%]	31; 20.3% [14.7%, 27.3%]
Only in hospital outpatients	125; 19.6% [16.7, 22.9]	88; 18.1% [15.0%, 21.8%]	37; 24.2% [18.1%, 31.5%]
In any of the above (all settings)	>538; 84.3% [81.3, 86.9]	390; 80.4% [76.6%, 83.7%]	>148; 96.7% [92.6%, 98.6%]

NCISH, National Confidential Inquiry into Suicide and Safety in Mental Health; SID-Cymru, Suicide Information Database – Wales; WLGP, Welsh Longitudinal General Practice Dataset; PEDW, Patient Episode Dataset for Wales; OPDW, Outpatient Database for Wales

We compared diagnostic information between NCISH and SAIL data for cases with primary care and hospital admission data in the year before their death (*n* = 755; demographics shown in Supplementary Table 4); out-patient diagnostic information was too scant to utilise. Over three-quarters of each primary NCISH diagnosis was recorded in SAIL data, except for personality disorder (62.5%) and adjustment disorder/reaction (only 25.0%). Comparing all NCISH-recorded diagnoses (not only primary diagnoses), NCISH and SAIL data mostly enhanced each other, with <50% of diagnoses recorded in both data sources in most instances. SAIL data generally provided more unique information than NCISH. Full results are shown in Supplementary Table 5.

### Contacts with services

We matched the 1031 cases in the final study cohort to 5155 controls (five per case) with virtually identical demographic distribution (Supplementary Table 3). Because of variable data availability across individual data-sets within the SAIL Databank, not all cases had data across all settings covering the year before the index date (Fig. 1 and Supplementary Fig. 1). Thus, the analysis of variables from each setting or combination of settings was based on cases with the required data coverage. The smallest subgroup was that with data across all settings (primary care, emergency department, and hospital admissions and out-patients), containing 34.4% (*n* = 355) of all cases. The full study population and all subgroups had similar demographic profiles, so it was reasonably assumed that data availability differences did not affect representativeness. Demographic details of each subcohort are shown in Supplementary Table 4.

Use of healthcare services by cases and controls in the year before the index date can be seen in Table 2. In the week before the index date, 42.5% of cases and 40.7% of controls had a healthcare service contact. The most common point of contact was primary care. Emergency department contacts and emergency hospital admissions were more common in cases than controls. One in six cases had emergency department contacts and emergency hospital admissions in the month before their index date, and almost two in three in the year before. Out-patient attendances were more common in controls than cases, and cases were less likely to have missed an out-patient appointment in the year before the index date.

**Table 2:** Type of health setting contacted before the index date

	Cases, *n*; % [95% CI]	Controls, *n*; % [95% CI]	Single-window regression, odds ratio [95% CI]; *P*-value	Multi-window regression, odds ratio [95% CI]; *P*-value
Any healthcare setting				
Subtotal	355 (100%)	1775 (100%)		
1 week	151; 42.5% [37.5–47.7%]	723; 40.7% [38.5–43.0%]	1.1 [0.9–1.2]; *P* = 0.275	0.3 [0.1–0.6]; *P* = 0.003
1 month	282; 79.4% [74.9–83.3%]	1468; 82.7% [80.9–84.4%]	0.8 [0.7–0.9]; *P* = 0.009	0.3 [0.1–0.5]; *P* < 0.001
1 year	349; 98.3% [96.4–99.2%]	1766; 99.5% [99.0–99.7%]	0.3 [0.1–0.6]; *P* = 0.002	0.3 [0.2–0.7]; *P* = 0.006
Primary care				
Subtotal	755 (100%)	3775 (100%)		
1 week	269; 35.6% [32.3–39.1%]	1242; 32.9% [31.4–34.4%]	1.1 [1.0–1.3]; *P* = 0.011	0.8 [0.6–1.0]; *P* = 0.032
1 month	531; 70.3% [66.9–73.5%]	2806; 74.3% [72.9–75.7%]	0.8 [0.7–0.9]; *P* < 0.001	0.6 [0.5–0.8]; *P* < 0.001
1 year	713; 94.4% [92.6–95.9%]	3624; 96.0% [95.3–96.6%]	0.7 [0.6–0.9]; *P* = 0.002	0.8 [0.7–1.0]; *P* = 0.065
Emergency department				
Subtotal	469 (100%)	2345 (100%)		
1 week	33; 7.0% [5.1–9.7%]	35; 1.5% [1.1–2.1%]	5.1 [3.5–7.4]; *P* < 0.001	7.1 [4.8–10.5]; *P* < 0.001
1 month	84; 17.9% [14.7–21.6%]	129; 5.5% [4.6–6.5%]	3.8 [3.1–4.8]; *P* < 0.001	4.3 [3.3–5.7]; *P* < 0.001
1 year	277; 59.1% [54.6–63.4%]	891; 38.0% [36.1–40.0%]	2.4 [2.1–2.7]; *P* < 0.001	1.9 [1.7–2.2]; *P* < 0.001
Hospital admissions				
Subtotal	1031 (100%)	5155 (100%)		
Emergency admissions				
1 week	40; 3.9% [2.9–5.2%]	100; 1.9% [1.6–2.4%]	2.1 [1.6–2.6]; *P* < 0.001	2.5 [1.9–3.2]; *P* < 0.001
1 month	156; 15.1% [13.1–17.4%]	359; 7.0% [6.3–7.7%]	2.4 [2.1–2.7]; *P* < 0.001	2.8 [2.4–3.3]; *P* < 0.001
1 year	599; 58.1% [55.1–61.1%]	2477; 48.0% [46.7–49.4%]	1.5 [1.4–1.7]; *P* < 0.001	1.3 [1.2–1.4]; *P* < 0.001
Planned admissions				
1 week	<5; 0.5% [0.2–1.1%]	30; 0.6% [0.4–0.8%]	DNC	0.7 [0.4–1.2]; *P* = 0.148
1 month	26; 2.5% [1.7–3.7%]	134; 2.6% [2.2–3.1%]	1.0 [0.8–1.2]; *P* = 0.802	1.1 [0.8–1.4]; *P* = 0.697
1 year	191; 18.5% [16.3–21.0%]	979; 19.0% [17.9–20.1%]	1.0 [0.9–1.1]; *P* = 0.544	0.9 [0.9–1.1]; *P* = 0.352
Hospital out-patient services				
Subtotal	848 (100%)	4240 (100%)		
Attended appointments				
1 week	42; 5.0% [3.7–6.6%]	244; 5.8% [5.1–6.5%]	0.9 [0.7–1.0]; *P* = 0.098	0.3 [0.2–0.4]; *P* < 0.001
1 month	131; 15.4% [13.2–18.0%]	915; 21.6% [20.4–22.8%]	0.7 [0.6–0.7]; *P* < 0.001	0.3 [0.2–0.3]; *P* < 0.001
1 year	462; 54.5% [51.1–57.8%]	3408; 80.4% [79.2–81.5%]	0.2 [0.2–0.3]; *P* < 0.001	0.2 [0.2–0.4]; *P* < 0.001
Missed or cancelled appointments				
1 week	24; 2.8% [1.9–4.2%]	112; 2.6% [2.2–3.2%]	1.1 [0.8–1.4]; *P* = 0.598	1.2 [0.9–1.6]; *P* = 0.152
1 month	60; 7.1% [5.5–9.0%]	370; 8.7% [7.9–9.6%]	0.8 [0.7–1.0]; *P* = 0.006	0.8 [0.7–1.0]; *P* = 0.105
1 year	298; 35.1% [32.0–38.4%]	2013; 47.5% [46.0–49.0%]	0.6 [0.5–0.6]; *P* < 0.001	0.9 [0.8–1.0]; *P* = 0.008

Numbers are counts, percentages and 95% confidence intervals of individuals. Odds ratios from conditional regression models for ‘any’ and each setting separately are shown. ‘Single-window regression’ models include a single dichotomous variable (1 week, 1 month or 1 year), whereas ‘multi-window regression’ models include a categorical variable encompassing all three time-windows in a non-overlapping way. Specified models did not converge (DNC) because of small numbers.

Across cases and controls, slightly more females than males had contacts with healthcare services, but both genders showed a similar pattern overall (Supplementary Table 6). The only notable exception was planned hospital admissions in the year before the index date, which was more common in female cases than controls (odds ratio 1.2, 95% CI 1.0–1.5), and less common in male cases than controls (odds ratio 0.8, 95% CI 0.7–0.9). We found no major differences in these variables between NCISH cases and SID-Cymru-only cases (see Supplementary Table 7).

The proportion of cases and controls with injury and poisoning, accidents and self-harm contacts in the year before the index date can be seen in Table 3. These contacts, particularly those for self-harm, and those in emergency department and hospital admissions, were more common in cases than controls. Across genders (Supplementary Table 8), 44.5% (95% CI 35.6–53.8%) of female cases had injury and poisoning contacts in the year before the index date, mostly hospital admissions (36.6%, 95% CI 31.6–41.9%). Odds ratios of injury and poisoning in hospital admissions changed substantially between the unadjusted (odds ratio 3.5, 95% CI 2.9–4.2) and adjusted models (odds ratio 1.6, 95% CI 1.0–2.3) in females only. We found no major differences on these variables between NCISH cases and SID-Cymru-only cases, except slightly elevated rates of emergency department contacts in SID-Cymru-only cases (see Supplementary Table 9).

**Table 3:** Accident, injury and poisoning, and self-harm contacts in the year before the index date

	Cases, *n*; % [95% CI]	Controls, *n*; % [95% CI]	Univariate regression, odds ratio [95% CI]; *P*-value	Multivariate regression, odds ratio [95% CI]; *P*-value
Any healthcare setting				
Subtotal	355 (100%)	1775 (100%)		
Accident	103; 29.0% [24.5–33.9%]	318; 17.9% [16.2–19.8%]	1.9 [1.6–2.2]; *P* < 0.001	1.3 [1.1–1.6]; *P* = 0.004
Injury and poisoning	134; 37.7% [32.9–41.0%]	331; 18.6% [16.9–20.5%]	2.8 [2.4–3.3]; *P* < 0.001	1.6 [1.3–1.9]; *P* < 0.001
Self-harm	101; 28.5% [24.0–33.4%]	150; 8.5% [7.2–9.8%]	4.6 [3.7–5.7]; *P* < 0.001	3.6 [2.8–4.5]; *P* < 0.001
Primary care				
Subtotal	755 (100%)	3775 (100%)		
Accident	27; 3.6% [2.5–5.2%]	122; 3.2% [2.7–3.8%]	1.1 [0.9–1.4]; *P* = 0.416	1.1 [0.8–1.4]; *P* = 0.497
Injury and poisoning	64; 8.5% [6.7–10.7%]	281; 7.4% [6.6–8.3%]	1.1 [1.0–1.4]; *P* = 0.103	1.0 [0.9–1.2]; *P* = 0.880
Self-harm	120; 15.9% [13.5–18.7%]	240; 6.4% [5.6–7.2%]	3.1 [2.6–3.7]; *P* < 0.001	3.1 [2.6–3.7]; *P* < 0.001
Emergency department				
Subtotal	469 (100%)	2345 (100%)		
Accident	100; 21.3% [17.9–25.3%]	366; 15.6% [14.2–17.1%]	1.5 [1.3–1.7]; *P* < 0.001	1.3 [1.1–1.6]; *P* = 0.002
Injury and poisoning	66; 14.1% [11.2–17.5%]	232; 9.9% [8.7–11.2%]	1.5 [1.3–1.8]; *P* < 0.001	1.2 [1.0–1.5]; *P* = 0.053
Self-harm	76; 16.2% [13.1–19.8%]	133; 5.7% [4.8–6.7%]	3.1 [2.5–3.8]; *P* < 0.001	3.0 [2.5–3.7]; *P* < 0.001
Hospital admissions				
Subtotal	1031 (100%)	5155 (100%)		
Accident	87; 8.4% [6.9–10.3%]	265; 5.1% [4.6–5.8%]	1.7 [1.5–2.0]; *P* < 0.001	0.8 [0.6–1.0]; *P* = 0.096
Injury and poisoning	307; 29.8% [27.1–32.6%]	630; 12.3% [11.4–13.1%]	3.2 [2.8–3.5]; *P* < 0.001	2.2 [1.8–2.8]; *P* < 0.001
Self-harm	225; 21.8% [19.4–24.4%]	360; 7.0% [6.3–7.7%]	3.9 [3.4–4.5]; *P* < 0.001	1.9 [1.5–2.4]; *P* < 0.001

Numbers are counts, percentages and 95% confidence intervals of individuals. Odds ratios from univariate and multivariate (i.e. including accident, injury and poisoning, and self-harm variables) conditional logistic regression models for ‘any’ and each healthcare setting separately are shown.

Of the 349 cases with fully linked data and who were in contact with healthcare services in the year before the index date, the last point of contact was primary care for 73.9% (*n* = 258), emergency department for 10.0% (*n* = 35), emergency hospital admission for 9.7% (*n* = 34), elective hospital admission for 1.7% (*n* = 6) and a hospital out-patients appointment for 1.7% (*n* = 6). During this last contact, injury and poisoning was recorded in 5.5% (*n* = 19) of cases, accident in 2.9% (*n* = 10) and self-harm in 6.0% (*n* = 21). Of the 77 admitted to hospital and/or seen as out-patients, 63.6% (*n* = 49) were seen by mental health services.

## Discussion

We found that those who died by suicide within 12 months of being in contact with mental health services (cases) had high rates of contacts in the week before their death across all settings: one in three had a primary care contact in the week before their death. When comparing these patients with those who did not die by suicide (controls), cases were more likely to attend the emergency department and be admitted to hospital with an emergency (almost two in three cases had such contacts in the year before the index date). Conversely, those who died by suicide in the year following contact with mental health services were seen less in primary care and as hospital out-patients than similar patients, despite lower levels of missed or cancelled out-patient appointments.

Over one in four mental health patients who died by suicide presented to healthcare services with accidents, injury and poisoning, and particularly self-harm in the year before their death. These presentations were less common in similar mental health patients who did not die by suicide, again particularly for self-harm. It may be that in those who died by suicide, presentations with accidents, injury and poisoning are likely undisclosed, misidentified or unrecorded self-harm events. Although odds ratios of self-harm contacts were similar between females and males overall (in line with the literature),^11,14^ they were higher in males for the emergency department and in females for hospital admission. Females also had higher odds ratios of contacts with injury and poisoning of undetermined or unspecified intent and, to a lesser extent (e.g. with overlapping confidence intervals), accidents, compared with males. We have previously reported this pattern across the whole population of suicide deaths,^20^ and it reinforces the suggestion of misidentified, non-disclosed or unrecorded self-harm events. Indeed, we have previously shown that some clinical codes for injury and poisoning of undetermined intent, such as rifle shotgun and larger firearm discharges, are highly indicative of self-harm;^40^ here, we have used these to identify self-harm and excluded them from injury and poisoning (Supplementary Table 2). The fact that odds ratios for hospital admissions with injury and poisoning changed drastically between the unadjusted to adjusted models, whereas those for self-harm changed the least, further indicates an important correlation between the two. The high rates of self-harm contacts in people who died by suicide within 12 months of being in contact with mental health services confirms that assessment and management of self-harm in line with NICE guidance is key to managing suicide risk.^10^

Despite these high rates of contacts with services, many who die by suicide within 12 months of being in contact with mental health services are assessed as low suicide risk by practitioners.^8^ We found similar rates of primary and emergency department contacts before suicide, as reported elsewhere.^8,9,11^ These contacts represent opportunities to intervene in primary and secondary care. Higher rates of accessing emergency care (emergency department, emergency hospital admission) in these patients (compared with mental health patients who did not die by suicide) are potentially an indicator of severity and/or a crisis leading to suicide, and this should therefore be considered when undertaking assessments. The lower rate of contacts with routine care (primary care, out-patients) often indicates poorer access to services, disengagement with care and lack of or non-adherence to any treatment. Other studies have found reduced prescribing and/or non-adherence to treatment in this cohort before suicide.^7,13,14^ However, we also found lower levels (small effect size) of missed or cancelled appointments in cases compared with controls, suggesting similar engagement in those referred to out-patient services. Overall, access and/or help-seeking issues earlier in the non-urgent care pathway (i.e. to primary care and out-patient services) warrant further investigation and should be addressed.^42^

By linking clinical audit data (NCISH data) with routinely collected data (SID-Cymru and data in the SAIL Databank), we identified an additional 307 suicide deaths in Wales (2001–2015) in contact with mental health services in the year before death that were missing from NCISH, amounting to over 20% of the total 1398 identified suicide cases. This discrepancy may be a result of differences in the definition of secondary care/specialist mental health service contacts (e.g. NCISH does not include one-off consultations with liaison psychiatry services/gateway services or contacts with a mental health nurse in primary care), and in the method and timing (i.e. delays) of recording contacts with mental health services. In contrast, routinely collected data alone would have missed around 15% of all the identified suicide cases (20% of cases identified by NCISH). Routinely collected data also allowed for inclusion of historical and psychiatric comorbidity information over and above clinical audit data. NCISH information is pertinent to mental health service contacts in the year before death, whereas SAIL contains all of the patient's health history inside and outside of mental health services. Finally, routinely collected data allowed us to identify matched controls with similar psychiatric diagnosis who were in contact with mental health services for comparison. Overall, our results highlight the power of linking clinical audit data and routinely collected data: the former provide detailed information, whereas the later add completeness and breath.

### Strengths and limitations

The linked clinical audit and routinely collected data covered the whole population of Wales (UK) over 15 years. Through this linkage, we identified an enhanced set of suicide cases and controls from the general population who were alive and also in contact with mental health services in the year before the matching case's death. To reduce bias, controls were matched on gender, week of birth, deprivation index, primary mental health diagnosis and similar data coverage. Deprivation index matching was key given the relatively high levels of deprivation and population sparsity in Wales compared with other parts of the UK,^43^ which are factors known to affect access to services and mental health outcomes. All of these allowed us to present a comprehensive description of the type and pattern of healthcare service contacts in the time leading up to death for cases, and compare this with controls.

Routinely collected data allowed us to identify additional (not in NCISH) suicide cases in contact with mental health services in the year before death. These may be part of the NCISH records not linked within SAIL, or may have been missed by the ONS reporting systems or by the mental health senior professional contacted by NCISH. A total of 32% of the additional cases had their mental health specialty contact recorded in SAIL only through a secondary care notification letter in primary care. These may be dated at the time of the secondary care contact or at the time the letter was received. If the latter, the contact with mental health services itself may have taken place outside of the year before death time window. In any case, our sensitivity analyses showed that cases identified in NCISH and those identified only in SID-Cymru were virtually identical with regards to the studied variables.

Although most published studies use death date as the index date,^14,15^ we replicated our previous, more robust, definition of index date,^20^ which excluded contacts during which the individual died. This is the date at which a patient's contact with healthcare services represented an opportunity to intervene. Nevertheless, we note that during a hospital admission, healthcare services have the opportunity to identify those at risk of suicide based on history of previous contacts.

The use of routinely collected data for research have several limitations reported elsewhere.^44,45^ For example, differences in the ascertainment of diagnoses between NCISH and SAIL could have played a role in the observed differences between the two data sources. The use of validated code lists to identify both our study cohort and outcomes of interest minimised misclassification bias to some extent; these were developed and updated with the help of expert clinicians. Missing variables and missing data biases was somewhat alleviated by the used case–control study design, as both groups experienced similar levels of ascertainment.

We could not guarantee that only face-to-face or telephone primary care contacts were included in the analysis, since secondary care and other contacts are communicated to primary care. Therefore, our primary care results may be inflated. In an attempt to alleviate this and maintain only telephone and face-to-face contacts with any member of the primary care team, we excluded full entries when they contained administrative codes such as ‘letter from emergency department’ or ‘patient seen in emergency department’.

We did not always have data for the full study population or study period across all settings. To circumvent this, we ran independent analyses with each of the data-sets (healthcare settings). We used several subpopulations based on data coverage limitations, but these had similar demographic distributions to the full population, suggesting that results were generalisable.

Finally, our analysis did not include other important factors. For example, we did not have access to data on referral route. Furthermore, we did not consider the order and geographical location of contacts across settings in the year before death. These and other factors should be considered by future research.

### Implications for policy and practice

Contrasting patterns of healthcare use in patients in contact with mental health services who did and did not die by suicide offer a unique insight and opportunity to identify better strategies for suicide prevention in this important population. We may be missing existing opportunities to intervene across all settings, particularly when people present to emergency departments and hospitals, especially with self-harm. Intent underlying injury and poisoning events may be undisclosed, or recorded as undetermined or without specifying intent when they may be self-harm, particularly in females. Efforts should be made to appropriately identify those who are self-harming, including by using direct and non-judgmental questioning on presentation, underpinned by staff training and awareness. Future research should qualitatively explore reasons for non-disclosure and opportunities to address identified barriers. Prevention efforts should focus on strengthening non-urgent and routine contacts (primary care and out-patient services), responding to emergency contacts and better self-harm care. This study also highlights the benefits of enhancing clinical audit systems with routinely collected data, for data completeness, breadth and depth.

## Supporting information

DelPozo-Banos et al. supplementary material 1 DelPozo-Banos et al. supplementary material

DelPozo-Banos et al. supplementary material 2 DelPozo-Banos et al. supplementary material

## Data Availability

Access to SAIL data is available on application to the SAIL Databank via their usage governance process (www.saildatabank.com). SID-Cymru data are available on request from the corresponding author (A.J.). NCISH data cannot be shared because of information governance restrictions in place to protect confidentiality. A request to access data can be made to the Healthcare Quality Improvement Partnership (www.hqip.org.uk/national-programmes/accessing-ncapop-data/).

## References

[1] Chung DT, Ryan CJ, Hadzi-Pavlovic D, Singh SP, Stanton C, Large MM. Suicide rates after discharge from psychiatric facilities: a systematic review and meta-analysis. JAMA Psychiatry 2017; 74(7): 694–702. 28564699 10.1001/jamapsychiatry.2017.1044 PMC5710249

[2] Bojanić L, Hunt IM, Baird A, Kapur N, Appleby L, Turnbull P. Early post-discharge suicide in mental health patients: findings from a national clinical survey. Front Psychiatry 2020; 11: 502. 10.3389/fpsyt.2020.00502 PMC7296132 32581877

[3] National Confidential Inquiry into Suicide and Safety in Mental Health. National Confidential Inquiry into Suicide and Safety in Mental Health Annual Report 2023: UK Patient and General Population Data 2010–2020. University of Manchester, 2023 (https://sites.manchester.ac.uk/ncish/reports/annual-report-2023/).

[4] National Confidential Inquiry into Suicide and Safety in Mental Health. National Confidential Inquiry into Suicide and Safety in Mental Health Annual Report 2022: UK Patient and General Population Data 2009–2019, and Real-Time Surveillance Data. University of Manchester, 2022 (https://nspa.org.uk/resource/national-confidential-inquiry-into-suicide-and-safety-in-mental-health-annual-report-2022-uk-patient-and-general-population-data-2009-2019-and-real-time-surveillance-data/).

[5] NHS England. Commissioning for Quality and Innovation (CQUIN): Guidance for 2020–2021. Publishing Approval Reference Number 001361. NHS England and NHS Improvement, 2019 (https://www.england.nhs.uk/wp-content/uploads/2020/01/FINAL-CQUIN-20-21-Core-Guidance-190220.pdf).

[6] National Institute for Health and Care Excellence (NICE). *Transition between Inpatient Mental Health Settings and Community or Care Home Settings*. NICE Guideline [NG53]. NICE, 2016 (https://www.nice.org.uk/guidance/ng53).

[7] Gianatsi M, Burns H, Hunt IM, Ibrahim S, Windfuhr K, While D, et al. Treatment of mental illness prior to suicide: a national investigation of 12,909 patients, 2001–2016. Psychiatr Serv 2020; 71(8): 772–8. 32340596 10.1176/appi.ps.201900452

[8] Pearson A, Saini P, Da Cruz D, Miles C, While D, Swinson N, et al. Primary care contact prior to suicide in individuals with mental illness. Br J Gen Pract 2009; 59(568): 825–32. 19861027 10.3399/bjgp09X472881 PMC2765834

[9] Da Cruz D, Pearson A, Saini P, Miles C, While D, Swinson N, et al. Emergency department contact prior to suicide in mental health patients. Emerg Med J 2011; 28(6): 467–71. 20660941 10.1136/emj.2009.081869

[10] National Institute for Health and Care Excellence (NICE). *Self-Harm: Assessment, Management and Preventing Recurrence*. *NICE Guideline [NG225]*. NICE, 2022 (https://www.nice.org.uk/guidance/ng225). 36595613

[11] Yim PH, Yip PS, Li RH, Dunn EL, Yeung WS, Miao YK. Suicide after discharge from psychiatric inpatient care: a case-control study in Hong Kong. Aust N Z J Psychiatry 2004; 38(1–2): 65–72. 14731196 10.1177/000486740403800103

[12] Yew Thong J, Hc Su A, Huak Chan Y, Hock Chia B. Suicide in psychiatric patients: case–control study in Singapore. Aust N Z J Psychiatry 2008; 42(6): 509–19. 18465378 10.1080/00048670802050553

[13] Troister T, Links PS, Cutcliffe J. Review of predictors of suicide within 1 year of discharge from a psychiatric hospital. Curr Psychiatry Rep 2008; 10(1): 60–5. 18269896 10.1007/s11920-008-0011-8

[14] Hunt IM, Kapur N, Webb R, Robinson J, Burns J, Shaw J, et al. Suicide in recently discharged psychiatric patients: a case-control study. Psychol Med 2009; 39(3): 443–9. 18507877 10.1017/S0033291708003644

[15] Bickley H, Hunt IM, Windfuhr K, Shaw J, Appleby L, Kapur N. Suicide within two weeks of discharge from psychiatric inpatient care: a case-control study. Psychiatr Serv 2013; 64(7): 653–9. 23545716 10.1176/appi.ps.201200026

[16] Meehan J, Kapur N, Hunt IM, Turnbull P, Robinson J, Bickley H, et al. Suicide in mental health in-patients and within 3 months of discharge: national clinical survey. Br J Psychiatry 2006; 188(2): 129–34. 16449699 10.1192/bjp.188.2.129

[17] Riblet N, Richardson JS, Shiner B, Peltzman TR, Watts BV, McCarthy JF. Death by suicide in the first year after irregular discharge from inpatient hospitalization. Psychiatr Serv 2018; 69(9): 1032–5. 29852826 10.1176/appi.ps.201800024 PMC8788660

[18] Office for National Statistics (ONS). *Suicides in England and Wales, 2021 Registrations*. ONS, 2022 (https://www.ons.gov.uk/peoplepopulationandcommunity/birthsdeathsandmarriages/deaths/bulletins/suicidesintheunitedkingdom/2021registrations?ref=publicsquare.uk).

[19] John A, Dennis M, Kosnes L, Gunnell D, Scourfield J, Ford DV, et al. Suicide Information Database-Cymru: a protocol for a population-based, routinely collected data linkage study to explore risks and patterns of healthcare contact prior to suicide to identify opportunities for intervention. BMJ Open 2014; 4(11): e006780. 10.1136/bmjopen-2014-006780 PMC4248097 25424996

[20] John A, DelPozo-Banos M, Gunnell D, Dennis M, Scourfield J, Ford DV, et al. Contacts with primary and secondary healthcare prior to suicide: case–control whole-population-based study using person-level linked routine data in Wales, UK, 2000–2017. Br J Psychiatry 2020; 217(6): 717–24. 32744207 10.1192/bjp.2020.137 PMC7705668

[21] Ford DV, Jones KH, Verplancke JP, Lyons RA, John G, Brown G, et al. The SAIL databank: building a national architecture for e-health research and evaluation. BMC Health Serv Res 2009; 9: 157. 19732426 10.1186/1472-6963-9-157 PMC2744675

[22] Lyons RA, Jones KH, John G, Brooks CJ, Verplancke JP, Ford DV, et al. The SAIL databank: linking multiple health and social care datasets. BMC Med Inform Decis Mak 2009; 9: 3. 19149883 10.1186/1472-6947-9-3 PMC2648953

[23] Welsh Government. Welsh Index of Multiple Deprivation (WIMD) 2014 Revised. Welsh Government, 2017 (https://www.gov.wales/sites/default/files/statistics-and-research/2019-04/welsh-index-of-multiple-deprivation-2014-revised.pdf).

[24] Barham C, Begum N. Analysis in brief-the new urban/rural indicator in the labour force survey-considers the potential uses of the rural and urban area classification 2004 in a labour market context. Labour Market Trends 2006; 114(12): 409.

[25] NSH Digital. *Read Codes*. NHS Digital, 2023 (https://digital.nhs.uk/services/terminology-and-classifications/read-codes).

[26] World Health Organization (WHO). *International Statistical Classification of Diseases and Related Health Problems 10th Revision (ICD-10)*. WHO, 2016 (https://icd.who.int/browse10/2016/en).

[27] Cornish RP, John A, Boyd A, Tilling K, Macleod J. Defining adolescent common mental disorders using electronic primary care data: a comparison with outcomes measured using the CIS-R. BMJ Open 2016; 6(12): e013167. 10.1136/bmjopen-2016-013167 PMC5168670 27909036

[28] Doyle M, While D, Mok PL, Windfuhr K, Ashcroft DM, Kontopantelis E, et al. Suicide risk in primary care patients diagnosed with a personality disorder: a nested case control study. BMC Fam Pract 2016; 17: 106. 27495284 10.1186/s12875-016-0479-y PMC4974738

[29] Durkin I, Kearney M, O'siorain L. Psychiatric disorder in a palliative care unit. Palliat Med 2003; 17(2): 212–8. 12701854 10.1191/0269216303pm670oa

[30] Ford E, Rooney P, Oliver S, Hoile R, Hurley P, Banerjee S, et al. Identifying undetected dementia in UK primary care patients: a retrospective case-control study comparing machine-learning and standard epidemiological approaches. BMC Med Inform Decis Making 2019; 19(1): 248. 10.1186/s12911-019-0991-9 PMC6889642 31791325

[31] Gradus JL, Qin P, Lincoln AK, Miller M, Lawler E, Lash TL. The association between adjustment disorder diagnosed at psychiatric treatment facilities and completed suicide. Clin Epidemiol 2010; 2: 23. 20865099 10.2147/clep.s9373 PMC2943177

[32] John A, Marchant AL, Fone DL, McGregor JI, Dennis MS, Tan JOA, et al. Recent trends in primary-care antidepressant prescribing to children and young people: an e-cohort study. Psychol Med 2016; 46(16): 3315–27. 27879187 10.1017/S0033291716002099 PMC5122314

[33] John A, McGregor J, Jones I, Lee SC, Walters JT, Owen MJ, et al. Premature mortality among people with severe mental illness—new evidence from linked primary care data. Schizophr Res 2018; 199: 154–62. 29728293 10.1016/j.schres.2018.04.009

[34] John A, Marchant A, Demmler J, Tan J, DelPozo-Banos M. Clinical management and mortality risk in those with eating disorders and self-harm: e-cohort study using the SAIL databank. BJPsych Open 2021; 7(2): e67. 10.1192/bjo.2021.23 PMC8058850 33736714

[35] John A, Friedmann Y, DelPozo-Banos M, Frizzati A, Ford T, Thapar A. Association of school absence and exclusion with recorded neurodevelopmental disorders, mental disorders, or self-harm: a nationwide, retrospective, electronic cohort study of children and young people in Wales, UK. Lancet Psychiatry 2022; 9(1): 23–34. 34826393 10.1016/S2215-0366(21)00367-9 PMC8674147

[36] Korkeila J, Oksanen T, Virtanen M, Salo P, Nabi H, Pentti J, et al. Early retirement from work among employees with a diagnosis of personality disorder compared to anxiety and depressive disorders. Eur Psychiatry 2011; 26(1): 18–22. 20541917 10.1016/j.eurpsy.2009.12.022

[37] Rees S, Watkins A, Keauffling J, John A. Incidence, mortality and survival in young people with co-occurring mental disorders and substance use: a retrospective linked routine data study in Wales. Clin Epidemiol 2022; 14: 21–38. 35058718 10.2147/CLEP.S325235 PMC8764170

[38] Underwood JF, DelPozo-Banos M, Frizzati A, John A, Hall J. Evidence of increasing recorded diagnosis of autism spectrum disorders in Wales, UK: an e-cohort study. Autism 2022; 26(6): 1499–508. 34841925 10.1177/13623613211059674 PMC9344561

[39] Wood S, Marchant A, Allsopp M, Wilkinson K, Bethel J, Jones H, et al. Epidemiology of eating disorders in primary care in children and young people: a clinical practice research datalink study in England. BMJ Open 2019; 9(8): e026691. 10.1136/bmjopen-2018-026691 PMC6688704 31378721

[40] Marchant A, Turner S, Balbuena L, Peters E, Williams D, Lloyd K, et al. Self-harm presentation across healthcare settings by sex in young people: an e-cohort study using routinely collected linked healthcare data in Wales, UK. Arch Dis Childhood 2020; 105(4): 347–54. 31611193 10.1136/archdischild-2019-317248 PMC7146921

[41] Newcombe RG. Two-sided confidence intervals for the single proposition: comparison of seven methods. Stat Med 1998; 17: 857–72. 9595616 10.1002/(sici)1097-0258(19980430)17:8<857::aid-sim777>3.0.co;2-e

[42] Troya MI, Chew-Graham CA, Babatunde O, Bartlam B, Mughal F, Dikomitis L. Role of primary care in supporting older adults who self-harm: a qualitative study in England. Br J Gen Pract 2019; 69(688): e740–51. 31594769 10.3399/bjgp19X706049 PMC6783141

[43] Abel GA, Barclay ME, Payne RA. Adjusted indices of multiple deprivation to enable comparisons within and between constituent countries of the UK including an illustration using mortality rates. BMJ Open 2016; 6(11): e012750. 10.1136/bmjopen-2016-012750 PMC5128942 27852716

[44] Benchimol EI, Smeeth L, Guttmann A, Harron K, Moher D, Petersen I, et al. The reporting of studies conducted using observational routinely-collected health data (RECORD) statement. PLoS Med 2015; 12(10): e1001885. 26440803 10.1371/journal.pmed.1001885 PMC4595218

[45] Pirkis J, Nicholas A, Gunnell D. The case for case–control studies in the field of suicide prevention. Epidemiol Psychiatr Sci 2020; 29: e62. 10.1017/S2045796019000581 PMC8061211 31571561

