# Supplementary material for: Contacts with primary and secondary healthcare before suicide by those under the care of mental health services: case–control, whole-population-based study using person-level linked routine data in Wales, UK during 2000–2015
Source: BJPsych Open. 2024 May 10;10(3):e108. doi: 10.1192/bjo.2024.23 (PMC11094447; doi:10.1192/bjo.2024.23)
Supplement: DelPozo-Banos et al. supplementary material 2 — DelPozo-Banos et al. supplementary material [file S2056472424000231sup002.pdf]

SUPPLEMENTARY MATERIAL

Contacts with primary and secondary healthcare before suicide by those under the care of mental health services: case-control whole-population-based study using person-level linked routine data in Wales, UK during 2000-2015

Marcos DelPozo-Banos[1], Cathryn Rodway[2], Sze Chim Lee[1], Olivier Y. Rouquette[1], Saied Ibrahim[2], Keith Lloyd[1], Louis Appleby[2], Nav Kapur[2,3,4], Ann John[1*]

* Corresponding author
[1] Swansea University Medical School, Wales, UK
[2] National Confidential Inquiry into Suicide and Safety in Mental Health (NCISH), Centre for Mental Health and Safety, School of Health Sciences, University of Manchester, Manchester, UK
[3] NIHR Greater Manchester Patient Safety Research Collaboration, University of Manchester, Manchester, UK
[4] Mersey Care NHS Foundation Trust, Prescot, UK

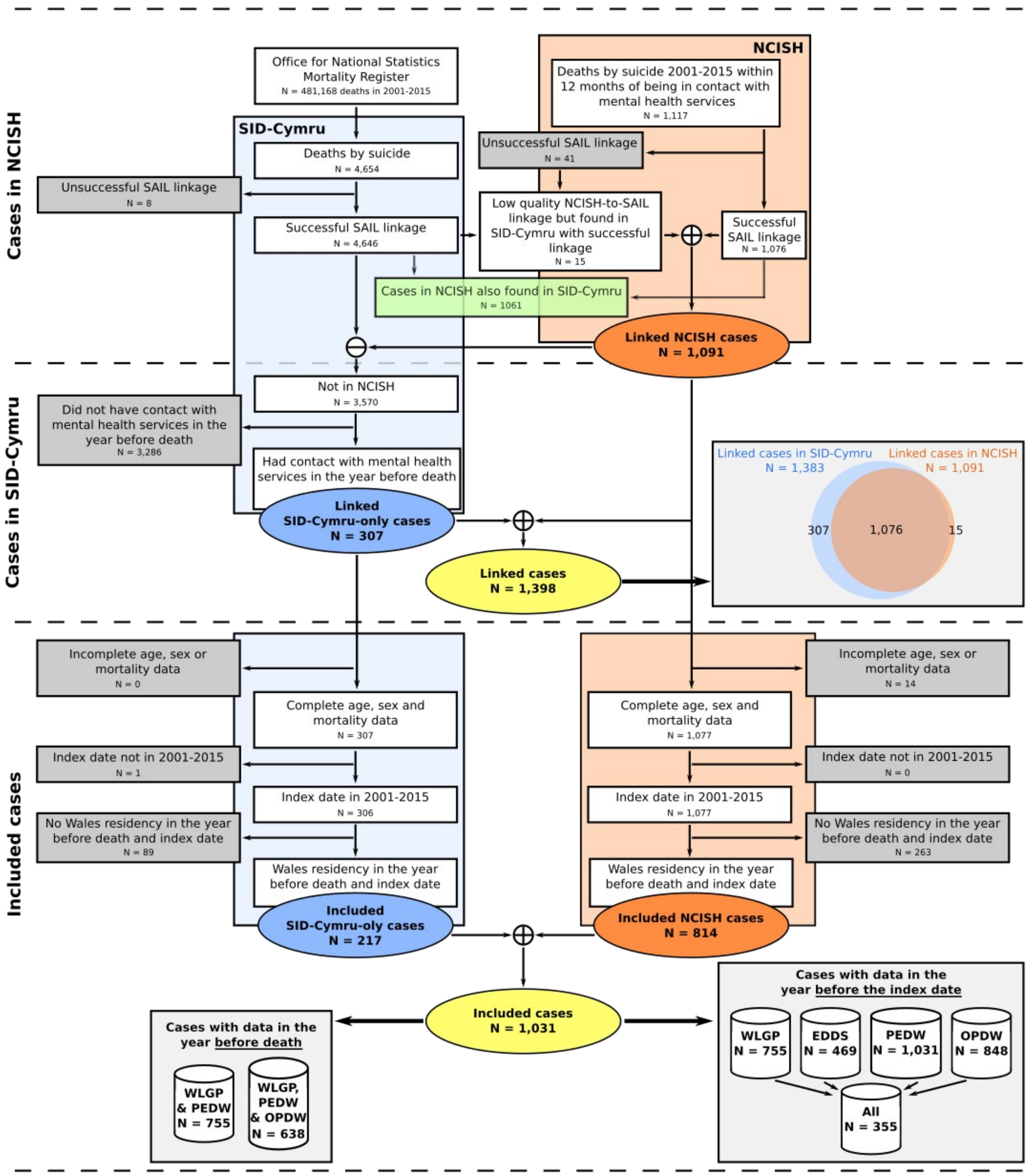

NCISH, National Confidential Inquiry into Suicide and Safety in Mental Health; SID-Cymru, Suicide Information Database – Wales; WLGP, Welsh Longitudinal General Practice Dataset; EDDS, Emergency Department Dataset; PEDW, Patient Episode Dataset for Wales; OPDW, Outpatient Database for Wales

Supplementary Figure 1. Detailed flow diagram of cases.

Supplementary Table 1: Details of the SAIL data sources used.

Name	Description	Coverage
Welsh Demographic Service	An administrative register of all individuals in Wales that use NHS services, containing anonymised demographics and GP practice registration history with anonymised residential data	The whole population of Wales during the study period
Office for National Statistics – Mortality Register	Death register of all deaths and causes in Wales, coded using International Classification of Diseases (ICD), version 10 codes	The whole population of Wales during the study period
Welsh Longitudinal General Practice Dataset, WLGP	Primary care records with diagnoses, symptoms, investigations, prescribed medication, referrals, coded hospital contacts, and test results coded using Read Codes v2	77% (333/432) of all general practices in Wales
Emergency Department Dataset, EDDS	Administrative and clinical information (general reason for attendance and attendance group to identify types of contacts) for all NHS Wales Accident and Emergency department attendances	The whole population of Wales from 2009
Patient Episode Dataset for Wales, PEDW	Clinical information (specialty and diagnoses) of all NHS Wales hospital admissions (inpatient and day cases) – diagnostic information coded using ICD-10 codes	The whole population of Wales during the study period
Outpatient Database for Wales, OPDW	Attendance information for all NHS Wales hospital outpatient appointments. Since diagnostic information was not available, we used consultant main specialty and treatment specialty to identify mental health contacts	The whole population of Wales from 2004

SAIL, Secure Anonymised Information Linkage Databank;

Supplementary Table 2: List and details of identified contacts with services across the Welsh Longitudinal General Practice Dataset (WLGP), the Emergency Department Dataset for Wales (EDDS), the Patient Episode Dataset for Wales (PEDW), and the Outpatients Database for Wales (OPDW).

Name	Description
Contacts	
Any contact	**WLGP**<br>Any entry excluding those with administrative codes and associated diagnoses such as 'letter from ED', but including telephone and face-to-face contacts with any member of the primary care team.<br><br>**EDDS, PEDW and OPDW**<br>Any entry.
Mental health specialty	**WLGP**<br>Records of psychiatric or child and adolescent psychiatric speciality recorded in WLGP (https://conceptlibrary.saildatabank.com/concepts/C3368/detail/).<br><br>**PEDW and OPDW**<br>Consultant main specialty, treatment specialty visited and hospital episodes with a mental health primary diagnosis – ICD-10 codes 'F'.
Diagnoses	
Any diagnoses	**WLGP**<br>Read codes with a capital letter root.
Mental health	**WLGP**<br>Read codes 'E'.<br><br>**EDDS**<br>Internal diagnostic code for mental health.<br><br>**PEDW**<br>ICD10 codes 'F'.
Schizophrenia and other delusional disorders*	**WLGP** [John2018]<br>https://conceptlibrary.saildatabank.com/concepts/C2716/version/6848/detail/<br><br>**PEDW** [John2018]<br>https://conceptlibrary.saildatabank.com/concepts/C2939/detail/
Bipolar affective disorder*	**WLGP** [John2018]<br>https://conceptlibrary.saildatabank.com/concepts/C2714/version/6844/detail/<br><br>**PEDW**<br>https://conceptlibrary.saildatabank.com/concepts/C2932/detail/
Eating disorder*	**WLGP** [Wood2019, John2021]<br>Read codes 'Eu500', 'Eu501', '1612', 'E271', 'R030'<br><br>**PEDW** [John2021]<br>ICD10 codes 'F50'
Dementia*	**WLGP** [Morgan2018; Ford2019]<br>Read codes 'E00', '1461', 'E012', 'E041', 'Eu00'-'Eu02', 'F110'-'F112', 'F116', 'F118', 'F11x'-'F11z', 'F134', 'E02y1', 'Eu02y', 'Eu02z', 'Eu041', 'Eu057', 'Fyu30', and 'Fyu30'.<br><br>**PEDW** [Morgan2018]<br>ICD10 codes 'F00'-'F03', 'G30', 'F051', and 'G311'.
Personality disorder*	**WLGP** [Doyle2016]<br>Read codes 'E21', 'Eu21', 'Eu60', 'Eu61', 'E02y4', 'Eu060', 'Eu340', 'Eu341', 'Eu845', 'Eu942'. Except codes 'E2121', 'E2122', 'E2140', 'E2141', 'E2151'-'E2153', 'Eu608'.<br><br>**PEDW** [Korkelia2011]<br>ICD-10 codes 'F60' and 'F61'.
Adjustment disorder/reaction*	**WLGP** [Morgan2018]<br>Read codes 'Eu432'.<br><br>**PEDW** [Gradus2010]<br>ICD-10 codes 'F432'.
Organic disorder*	**WLGP**<br>Read codes 'Eu0' except those used for dementia [Morgan2018]<br><br>**PEDW**<br>ICD-10 codes 'F00'-'F07' and 'F09' [Durking2010] except those used for dementia [Morgan2018; Ford2019]
Learning disability*	**WLGP**<br>1j9, 69DB, 8Ce6, 8H4f, 8HHP, 8Hg2, 918e, 94Z9, 9HB, 9N0y, 9hL, 9mA, Eu7, PJ0, E3, Eu814-Eu818<br><br>**PEDW**<br>F70-F73, F78-F79, F819, F84 (except F84.0, F84.1, F84.5), Q90-Q93, Q95.2-Q95.9, Q97.0, Q97.1, Q98.1, Q99.2, Q99.8,
Pervasive development disorder/autistic spectrum disorder*	**WLGP** [Underwood2021]<br>https://conceptlibrary.saildatabank.com/concepts/C2709/version/6834/detail/<br><br>**PEDW** [Underwood2021]<br>ICD-10 codes 'F840', 'F841', 'F845' and 'F849'
ADHD/conduct disorder*	**WLGP** [John2022]<br>https://conceptlibrary.saildatabank.com/concepts/C2708/version/6832/detail/<br><br>**PEDW** [John2022]

	ICD-10 codes ‘F90’
Somatoform/somatisation disorder*	**WLGP**<br>Read codes ‘Eu45’<br><br>**PEDW**<br>ICD-10 codes ‘F45’
Depression*	**WLGP** [John2016, Cornish2016]<br>https://conceptlibrary.saildatabank.com/concepts/C3295/version/10153/detail/<br>Includes contacts with a recorded depression diagnosis, and contacts with a recorded antidepressant treatment or prescription following a previous depression diagnosis. Administrative codes that unequivocally indicate a diagnosis of depression are used as "depression diagnoses" by the algorithm.<br><br>**PEDW** [John2016, Cornish2016]<br>https://conceptlibrary.saildatabank.com/concepts/C2942/version/7736/detail/
Anxiety*	**WLGP** [John2016, Cornish2016]<br>https://conceptlibrary.saildatabank.com/concepts/C3297/version/10127/detail/<br>Includes contacts with a recorded anxiety diagnosis or symptom, and contacts with a recorded anxiolytic or hypnotic prescription following a previous anxiety diagnosis. Therapeutic procedures that unequivocally indicate a diagnosis of anxiety are used as "anxiety diagnoses" by the algorithm.<br><br>**PEDW** [John2016, Cornish2016]<br>https://conceptlibrary.saildatabank.com/concepts/C3297/version/10127/detail/
Injury & poisoning	**WLGP**<br>Read codes ‘S’, ‘T’, ‘U’ Except: ‘SL’ (single code), ‘U72’ (single code), ‘SL90’, ‘SLHz’, ‘U720’ and ‘SS’ and their child codes.<br><br>**EDDS**<br>Attendances with any injury attendance groups except those identified as self-harm [Marchant2020].<br><br>**PEDW**<br>ICD-10 codes ‘S00’-’S99’ and ‘T00’-’T98’.
Accidents	**WLGP**<br>Read codes ‘SS’, ‘T0’-’T9’, ‘TA’-’TH’, ‘TJ’, ‘U0’, ‘U1’<br><br>**EDDS**<br>Recorded attendance group ‘accident’.<br><br>**PEDW**<br>ICD-10 codes ‘V’, ‘W’, ‘X1’-’X5’
Self-harm	**WLGP** [Marchant2020]<br>https://conceptlibrary.saildatabank.com/concepts/C3292/detail/<br><br>**EDDS** [Marchant2020]<br>Recorded attendance group ‘deliberate SH’ and attendance group of ‘undetermined intent’ when presenting with lacerations to wrist or forearm; and overdose or poisoning by prescribed drugs, non-prescribed/purchased drugs, elicit drugs, and unspecified. Codes referring to self-harm with alcohol were excluded.<br><br>**PEDW** [Marchant2020]<br>https://conceptlibrary.saildatabank.com/concepts/C3292/detail/
Alcohol misuse*	**WLGP** [Rees2022]<br>https://conceptlibrary.saildatabank.com/concepts/C3293/detail/<br><br>**EDDS**<br>Internal diagnostic codes for alcohol poisoning and overdose and chronic alcohol abuse.<br><br>**PEDW** [Rees2022]<br>https://conceptlibrary.saildatabank.com/concepts/C3293/detail/
Drugs misuse*	**WLGP** [Rees2022]<br>https://conceptlibrary.saildatabank.com/concepts/C3294/detail/<br><br>**EDDS**<br>Internal diagnostic codes for poisoning and overdose by illicit drug and chronic drug abuse.<br><br>**PEDW** [Rees2022]<br>https://conceptlibrary.saildatabank.com/concepts/C3294/detail/
Prescriptions (**recorded on WLGP only**)	
Any prescription	Read code with a lower case letter root
Opiates	Read codes 8B23, 8B2M, 8B2N, 8B2P, 8B2Q, 8B2R, 8BE, a81, cg1, cg3, cg5, cg7, dia, dib, dic, did6, dj, j28O, j28V, j2pT, o4, oa2N, and oa2J, except dia8A-diaF, diaH, diaM, diaN, diaQ, diaR, diaV-diaZ, diaa, diac, diaf, diag, diaj, dial, diau. diay, diaz, dib1, dib2, dib4-dib7, dibC, dibL, dibN, dibO, dibP, dibT, dibU, dibV, dibY, dibZ, dibi, dibk, dibl, dibm, dibq, dibr, diby, dic1, dic2, dic6, dicB-dicI, dicP, dicQ, dicT-dicX, dicZ, dicw, djm, o43, o4a, cg7c, dj3K, dj3L, dj3M, djkb, djkc, djkd, djyJ, djyK, djyL, o4e4-o4e8
Psychotropics	Includes Read codes from antidepressants, antipsychotics, anxiolytics, and hypnotics (see below)
Antidepressants	https://conceptlibrary.saildatabank.com/concepts/C2917/version/7757/detail/
Antipsychotics	Read codes d4, d5
Anxiolytics	https://conceptlibrary.saildatabank.com/concepts/C2924/version/7710/detail/
Hypnotics	https://conceptlibrary.saildatabank.com/concepts/C2925/version/7711/detail/
Lithium	Read codes d6

Unless otherwise specified (“single code”), when a Read code or an ICD-10 code is included, all its child codes are also included.

* Diagnosis available in NCISH data.

[Cornish2016] Cornish, R. P., John, A., Boyd, A., Tilling, K., & Macleod, J. (2016). Defining adolescent common mental disorders using electronic primary care data: A comparison with outcomes measured using the CIS-R. BMJ Open, 6(12). https://doi.org/10.1136/bmjopen-2016-013167

[Doyle2016] Doyle, M., While, D., Mok, P. L., Windfuhr, K., Ashcroft, D. M., Kontopantelis, E., ... & Webb, R. T. (2016). Suicide risk in primary care patients diagnosed with a personality disorder: a nested case control study. BMC family practice, 17(1), 1-9.

[Durkin2010] Durkin, I., Kearney, M., & O'siorain, L. (2003). Psychiatric disorder in a palliative care unit. Palliative medicine, 17(2), 212-218.

[Ford2019] Ford, E., Rooney, P., Oliver, S., Hoile, R., Hurley, P., Banerjee, S., ... & Cassell, J. (2019). Identifying undetected dementia in UK primary care patients: a retrospective case-control study comparing machine-learning and standard epidemiological approaches. BMC medical informatics and decision making, 19(1), 1-9.

[Gradus2010] Gradus, J. L., Qin, P., Lincoln, A. K., Miller, M., Lawler, E., & Lash, T. L. (2010). The association between adjustment disorder diagnosed at psychiatric treatment facilities and completed suicide. Clinical epidemiology, 2, 23.

[Marchant2020] Marchant, A., Turner, S., Balbuena, L., Peters, E., Williams, D., Lloyd, K., ... & John, A. (2020). Self-harm presentation across healthcare settings by sex in young people: an e-cohort study using routinely collected linked healthcare data in Wales, UK. Archives of disease in childhood, 105(4), 347-354. https://doi.org/10.1111/bcp.12059

[Morgan2018] Morgan, C., Webb, R. T., Carr, M. J., Kontopantelis, E., Chew-Graham, C. A., Kapur, N., & Ashcroft, D. M. (2018). Self-harm in a primary care cohort of older people: incidence, clinical management, and risk of suicide and other causes of death. The Lancet Psychiatry, 5(11), 905-912.

[John2016] John, A., Marchant, A. L., Fone, D. L., McGregor, J. I., Dennis, M. S., Tan, J. O. A., & Lloyd, K. (2016). Recent trends in primary-care antidepressant prescribing to children and young people: an e-cohort study. Psychological Medicine, 46(16), 3315–3327. https://doi.org/10.1017/S0033291716002099

[John2018] John, A., McGregor, J., Jones, I., Lee, S. C., Walters, J. T., Owen, M. J., ... & Lloyd, K. (2018). Premature mortality among people with severe mental illness—New evidence from linked primary care data. Schizophrenia research, 199, 154-162.

[John2021] John, A., Marchant, A., Demmler, J., Tan, J., & DelPozo-Banos, M. (2021). Clinical management and mortality risk in those with eating disorders and self-harm: e-cohort study using the SAIL databank. BJPsych open, 7(2).

[John2022] John, A., Friedmann, Y., DelPozo-Banos, M., Frizzati, A., Ford, T., & Thapar, A. (2022). Association of school absence and exclusion with recorded neurodevelopmental disorders, mental disorders, or self-harm: a nationwide, retrospective, electronic cohort study of children and young people in Wales, UK. The Lancet Psychiatry, 9(1), 23-34.

[Korkeila2011] Korkeila, J., Oksanen, T., Virtanen, M., Salo, P., Nabi, H., Pentti, J., ... & Kivimäki, M. (2011). Early retirement from work among employees with a diagnosis of personality disorder compared to anxiety and depressive disorders. European Psychiatry, 26(1), 18-22.

[Rees2022] Rees, S., Watkins, A., Keauffling, J., & John, A. (2022). Incidence, Mortality and Survival in Young People with Co-Occurring Mental Disorders and Substance Use: A Retrospective Linked Routine D

[Underwood2021] Underwood, J. F., DelPozo-Banos, M., Frizzati, A., John, A., & Hall, J. (2021). Evidence of increasing recorded diagnosis of autism spectrum disorders in Wales, UK: An e-cohort study. Autism, 13623613211059674.

[Wood2019] Wood, S., Marchant, A., Allsopp, M., Wilkinson, K., Bethel, J., Jones, H., & John, A. (2019). Epidemiology of eating disorders in primary care in children and young people: a clinical practice research Datalink study in England. BMJ open, 9(8), e026691.

Supplementary Table 3. Demographics of cases and controls in the study population (top half), and their mental health service contacts in the year before death for those with primary care, hospital admissions and hospital outpatient data in this period for the largest sub-cohort with the minimum required data sources (bottom half).

		Cases, N; % [95% CI]	Controls, N; % [95% CI]
Full study population	**Total**	**1031 (100%)**	**5155 (100%)**
	**Males/Females**		
	Females	328; 31.8% [29.0, 34.7]	1640; 31.8% [30.6, 33.1]
	Males	703; 68.19% [65.3, 71.0]	3515; 68.2% [66.9, 69.4]
	**Age groups**		
	[10, 24] years old	69; 6.7% [5.3, 8.4]	357; 6.93% [6.27, 7.66]
	[25, 64] years old	807; 78.3% [75.7, 80.7]	4029; 78.16% [77.01, 79.27]
	64+ years old	155; 15.0% [13.0, 17.3]	769; 14.92% [13.98, 15.92]
	**Deprivation index**		
	Least deprived - 1	158; 15.3% [13.3, 17.7]	783; 15.2% [14.2, 16.2]
	2	170; 16.5% [14.3, 18.9]	863; 16.7% [15.7, 17.8]
	3	205; 19.9% [17.6, 22.4]	1021; 19.8% [18.7, 20.9]
	4	246; 23.9% [21.4, 26.6]	1227; 23.8% [22.7, 25.0]
	Most deprived - 5	252; 24.4% [21.9, 27.2]	1261; 24.5% [23.3, 25.7]
	Missing value	<5; 0.5% [0.2, 1.1]	<5; 0.1% [0.0, 0.2]
	**Area morphology**		
	Urban	719; 69.7% [66.9, 72.5]	3527; 68.4% [67.1, 69.7]
	Rural	182; 17.7% [15.4, 20.1]	939; 18.2% [17.2, 19.3]
	Missing value	<5; 0.5% [0.2, 1.1]	<5; 0.1% [0.0, 0.2]
Study population with WLGP, PEDW and OPDW data in the year before death	**Total**	**638 (100%)**	**3190 (100%)**
	**Mental health service contacts in primary care data**		
	Recorded contact	245; 38.4% [37.7, 42.2]	1295; 40.6% [38.9, 42.3]
	**Mental health service contacts in hospital admissions data**		
	Specialty	238; 38.4% [34.7, 42.2]	926; 29.0% [27.5, 30.6]
	Health Resource Group	89; 13.9% [11.5, 16.9]	610; 19.1% [17.8, 20.5]
	Main diagnosis	207; 32.4% [28.9, 36.2]	964; 30.2% [28.7, 31.8]
	Any of the above	265; 41.5% [37.8, 45.4]	1162; 36.4% [34.8, 38.1]
	**Mental health service contacts in hospital outpatients data**		
	Specialty	305; 47.8% [44.0, 51.7]	2467; 77.3% [75.9, 78.8]
	**Mental health service contacts in recorded**		
	Only in primary care	94; 14.7% [12.2, 17.7]	<5; 0.2% [0.1, 0.4]
	Only in hospital admissions	105; 16.5% [13.8, 19.5]	531; 16.6% [15.4, 18.0]
	Only in hospital outpatients	125; 19.6% [16.7, 22.9]	1157; 36.3% [34.6, 38.0]
	**In any of the above (all settings)**	**>538; 84.3% [81.3, 86.9]**	**3190; 100%**

Numbers are counts, percentages and 95% confidence intervals of individuals.

Supplementary Table 4. Demographics of sub-cohorts of cases with different combinations of data sources available within the SAIL Databank in the year before death and before the index date.

	**Cases w. WLGP and PEDW data in the year before death (N; % [95% CI])**	**Cases w. WLGP, PEDW and OPD data in the year before death (N; % [95% CI])**	**Cases with WLGP data in the year before the index date**	**Cases with EDDS data in the year before the index date**	**Cases with PEDW data in the year before the index date**	**Cases with OPD data in the year before the index date**	**Cases with full data linkage in the year before the index date**
**Total**	**755 (100%)**	**638 (100%)**	**755 (100%)**	**469 (100%)**	**1031 (100%)**	**848 (100%)**	**355 (100%)**
**Males/Females**							
Females	249; 33.0% [29.7%, 36.4%]	206; 32.3% [28.8%, 36.0%]	249; 33.0% [29.7%, 36.4%]	139; 29.6% [25.7%, 33.9%]	328; 31.8% [29.0%, 34.7%]	269; 31.73% [28.68%, 34.94%]	110; 31.0% [26.4%, 36.0%]
Males	506; 67.0% [63.6%, 70.3%]	432; 67.7% [64.0%, 71.2%]	506; 67.0% [63.6%, 70.3%]	330; 70.4% [66.0%, 74.3%]	703; 68.2% [65.3%, 71.0%]	579; 68.28% [65.07%, 71.33%]	245; 69.0% [64.0%, 73.6%]
**Age groups**							
Children & young people: [10, 24] y.o.	49; 6.5% [4.9%, 8.5%]	39; 6.1% [4.5%, 8.2%]	49; 6.5% [4.95%, 8.48%]	26; 5.55% [3.82%, 8%]	69; 6.7% [5.33%, 8.39%]	52; 6.14% [4.71%, 7.96%]	20; 5.64% [3.68%, 8.55%]
Adults: [25, 64] y.o.	583; 77.2% [74.1%, 80.1%]	496; 77.7% [74.4%, 80.8%]	583; 77.22% [74.1%, 80.07%]	373; 79.54% [75.65%, 82.94%]	807; 78.28% [75.66%, 80.69%]	670; 79.01% [76.15%, 81.62%]	278; 78.31% [73.74%, 82.29%]
Older population: 64+ y.o.	123; 16.3% [13.8%, 19.1%]	103; 16.1% [13.5%, 19.2%]	123; 16.3% [13.83%, 19.1%]	70; 14.93% [11.99%, 18.44%]	155; 15.04% [12.99%, 17.35%]	126; 14.86% [12.63%, 17.42%]	57; 16.06% [12.61%, 20.24%]
**Deprivation index**							
Least deprived - 1	125; 16.6% [14.1%, 19.4%]	111; 17.4% [14.7%, 20.5%]	125; 16.6% [14.1%, 19.4%]	74; 15.8% [12.8%, 19.4%]	158; 15.3% [13.3%, 17.7%]	137; 16.2% [13.8%, 18.8%]	58; 16.3% [12.9%, 20.5%]
2	111; 14.7% [12.4%, 17.4%]	92; 14.4% [11.9%, 17.4%]	111; 14.7% [12.4%, 17.4%]	83; 17.7% [14.5%, 21.4%]	170; 16.5% [14.3%, 18.9%]	142; 16.7% [14.4%, 19.4%]	55; 15.5% [12.1%, 19.6%]
3	157; 20.8% [18.1%, 23.8%]	131; 20.5% [17.6%, 23.8%]	157; 20.8% [18.1%, 23.8%]	90; 19.2% [15.9%, 23.0%]	205; 19.9% [17.6%, 22.4%]	164; 19.3% [16.8%, 22.1%]	74; 20.8% [16.9%, 25.4%]
4	170; 22.5% [19.7%, 25.6%]	141; 22.1% [19.1%, 25.5%]	170; 22.5% [19.7%, 25.6%]	111; 23.7% [20.0%, 27.7%]	246; 23.9% [21.4%, 26.6%]	197; 23.2% [20.5%, 26.2%]	81; 22.8% [18.7%, 27.5%]
Most deprived - 5	192; 25.4% [22.5%, 28.7%]	163; 25.5% [22.3%, 29.1%]	192; 25.4% [22.5%, 28.7%]	111; 23.7% [20.0%, 27.7%]	252; 24.4% [21.9%, 27.2%]	208; 24.5% [21.8%, 27.5%]	87; 24.5% [20.3%, 29.2%]
Missing deprivation value	<5; 0.7% [0.3%, 1.5%]	<5; 0.8% [0.3%, 1.8%]	<5; 0.7% [0.3%, 1.5%]	<5; 1.1% [0.5%, 2.5%]	<5; 0.5% [0.2%, 1.1%]	<5; 0.6% [0.3%, 1.4%]	<5; 1.4% [0.6%, 3.3%]
**Area morphology**							
Urban	553; 73.2% [70.0%, 76.3%]	463; 72.6% [69.0%, 75.9%]	553; 73.2% [70.0%, 76.3%]	332; 70.8% [66.5%, 74.7%]	719; 69.7% [66.9%, 72.5%]	591; 69.7% [66.5%, 72.7%]	262; 73.8% [69.0%, 78.1%]
Rural	117; 15.5% [13.1%, 18.3%]	104; 16.3% [13.6%, 19.4%]	117; 15.5% [13.1%, 18.3%]	76; 16.2% [13.1%, 19.8%]	182; 17.7% [15.4%, 20.1%]	155; 18.3% [15.8%, 21.0%]	54; 15.2% [11.8%, 19.3%]
Missing rurality value	<5; 0.7% [0.3%, 1.5%]	<5; 0.8% [0.3%, 1.8%]	<5; 0.7% [0.3%, 1.5%]	<5; 1.1% [0.5%, 2.5%]	<5; 0.5% [0.2%, 1.1%]	<5; 0.6% [0.3%, 1.4%]	<5; 1.4% [0.6%, 3.3%]

Numbers are counts, percentages and 95% confidence intervals of individuals.
SAIL, Secure Anonymised Information Linkage Databank; WLGP, Welsh Longitudinal General Practice Dataset; EDDS, Emergency Department Dataset for Wales; PEDW, Patient Episode Dataset for Wales; OPDW, Outpatients Database for Wales.

Supplementary Table 5. Comparison of diagnostic information between NCISH and SAIL data sources for cases with primary care and hospital admission data (N = 755).

**Diagnosis**	**NCISH (N)**	**In SAIL (%)**	**NCISH or SAIL (N)**	**NCISH not SAIL (%)**	**NCISH & SAIL (%)**	**SAIL not NCISH(%)**
Schizophrenia and other delusional disorders	89	75.3%	**132**	16.7%	50.8%	32.6%
Bipolar affective disorder	53	84.9%	**83**	10.8%	55.4%	33.7%
Depressive illness	213	91.1%	**495**	5.7%	52.3%	42.0%
Anxiety/phobia/panic disorder/OCD	24	87.5%	**323**	5.6%	23.8%	70.6%
Eating disorder	6	83.3%	**12**	<50.0%	50.0%	<50.0%
Dementia	<5		**11**	<45.5%	<45.5%	>54.5%
Personality disorder	48	62.5%	**127**	36.2%	34.6%	29.1%
Adjustment disorder/reaction	28	25.0%	**82**	46.3%	12.2%	41.5%
Organic disord	<5		**18**	<27.8%	<27.8%	72.2%
Learning disability	<5		**<10**			
Pervasive development disorder/autistic spectrum disorder	<5		**<10**			
ADHD/conduct disorder	<5		**<10**			
Somatoform/somatisation disorder	<5		**<10**			
Alcohol dependence/misuse	49	>89.8%	**237**	11.8%	45.6%	42.6%
Drug dependence/misuse	29	>82.8%	**179**	13.4%	35.2%	51.4%

Columns are: number of cases with each primary diagnosis (NCISH) and the proportion of them with such diagnosis recorded in SAIL (In SAIL); number of cases with each diagnosis (NCISH or SAIL) and the proportion of them with such diagnosis recorded in NCISH only (NCISH not SAIL), both NCISH and SAIL (NCISH & SAIL), and SAIL only (SAIL not NCISH).

NCISH, National Confidential Inquiry into Suicide and Safety in Mental Health; SAIL, Secure Anonymised Information Linkage Databank

Supplementary Table 6 (part 1/2). Type of healthcare setting contacted before index date stratified by sex.

	Females cases (N; % [95% CI])	Females controls (N; % [95% CI])	Single-window regression (OR [95% CI]; p-value)	Multi-window regression (OR [95% CI]; p-value)
**Any healthcare setting**				
**Sub-total**	**110 (100%)**	**550 (100%)**		
1 week	53; 48.2% [39.1%, 57.4%]	273; 49.6% [45.5%, 53.8%]	1.0 [0.8, 1.2]; p=0.626	DNC
1 month	92; 83.6% [75.6%, 89.4%]	484; 88% [85%, 90.5%]	0.7 [0.5, 1.0]; p=0.034	DNC
1 year	>105; 95.5% [89.8%, 98.1%]	>545; 99.1% [97.9%, 99.6%]	DNC	DNC
**Primary care**				
**Sub-total**	**249 (100%)**	**1245 (100%)**		
1 week	102; 41.0% [35.0%, 47.2%]	485; 39.0% [36.3%, 41.7%]	1.1 [0.9, 1.3]; p=0.295	0.5 [0.3, 0.8]; p=0.005
1 month	185; 74.3% [68.5%, 79.3%]	995; 79.9% [77.6%, 82.1%]	0.7 [0.6, 0.9]; p=<0.001	0.4 [0.3, 0.7]; p=<0.001
1 year	236; 94.8% [91.3%, 96.9%]	1214; 97.5% [96.5%, 98.3%]	0.5 [0.3, 0.7]; p=<0.001	0.6 [0.4, 0.9]; p=0.023
**Emergency department**				
**Sub-total**	**139 (100%)**	**695 (100%)**		
1 week	12; 8.6% [5.0%, 14.5%]	18; 2.6% [1.7%, 4.1%]	3.8 [2.2, 6.7]; p=<0.001	6.8 [3.7, 12.5]; p=<0.001
1 month	27; 19.4% [13.7%, 26.8%]	47; 6.8% [5.1%, 8.9%]	3.5 [2.4, 5]; p=<0.001	4.9 [3.0, 8.0]; p=<0.001
1 year	95; 68.4% [60.2%, 75.5%]	273; 39.3% [35.7%, 43%]	3.4 [2.7, 4.3]; p=<0.001	2.9 [2.3, 3.8]; p=<0.001
**Hospital admissions**				
**Sub-total**	**328 (100%)**	**1640 (100%)**		
Emergency admissions				
1 week	22; 6.7% [4.5%, 10%]	36; 2.2% [1.6%, 3%]	3.3 [2.2, 4.8]; p=<0.001	4.6 [3.0, 6.8]; p=<0.001
1 month	53; 16.2% [12.6%, 20.5%]	119; 7.3% [6.1%, 8.6%]	2.5 [2.0, 3.1]; p=<0.001	2.7 [2.0, 3.6]; p=<0.001
1 year	215; 65.6% [60.3%, 70.5%]	839; 51.2% [48.7%, 53.6%]	1.9 [1.6, 2.2]; p=<0.001	1.6 [1.4, 1.9]; p=<0.001
Planned admissions				
1 week	<5; 1.5% [0.7%, 3.5%]	17; 1.0% [0.7%, 1.7%]	DNC	0.4 [0.1, 0.9]; p=0.038
1 month	9; 2.8% [1.5%, 5.1%]	56; 3.4% [2.6%, 4.4%]	0.8 [0.5, 1.2]; p=0.261	1.2 [0.7, 1.9]; p=0.516
1 year	77; 23.5% [19.2%, 28.4%]	327; 19.9% [18.1%, 22.0%]	1.2 [1.1, 1.5]; p=0.014	1.3 [1.0, 1.5]; p=0.022
**Hospital outpatients**				
Sub-total	269 (100%)	1345 (100%)		
Attended appointments				
1 week	19; 7.1% [4.6%, 10.8%]	85; 6.3% [5.1%, 7.8%]	1.1 [0.8, 1.5]; p=0.436	0.4 [0.2, 0.5]; p=<0.001
1 month	53; 19.7% [15.4%, 24.9%]	310; 23.1% [20.9%, 25.4%]	0.8 [0.7, 1.0]; p=0.029	0.3 [0.2, 0.3]; p=<0.001
1 year	162; 60.2% [54.3%, 65.9%]	1114; 82.8% [80.7%, 84.8%]	0.3 [0.2, 0.3]; p=<0.001	0.2 [0.2, 0.3]; p=<0.001
Missed or cancelled appointments				
1 week	7; 2.6% [1.3%, 5.3%]	44; 3.3% [2.5%, 4.4%]	0.8 [0.5, 1.2]; p=0.313	0.9 [0.6, 1.5]; p=0.737
1 month	23; 8.6% [5.8%, 12.5%]	136; 10.1% [8.6%, 11.8%]	0.8 [0.7, 1.1]; p=0.172	1.1 [0.7, 1.5]; p=0.806
1 year	112; 41.6% [35.9%, 47.6%]	658; 48.9% [46.3%, 51.6%]	0.7 [0.6, 0.9]; p=<0.001	1.1 [0.9, 1.4]; p=0.221

Numbers are counts, percentages and 95% confidence intervals of individuals. Odds ratios from conditional logistic regression models for 'any' and each setting separately are shown. 'Single-window regression' models include a single dichotomous variable (1 week, 1 month or 1 year), while 'multi-window regression' models include a categorical variable encompassing all three time-windows in a non-overlapping way. Specified models did not converged (DNC) due to small numbers.

Supplementary Table 6 (part 2/2). Type of health setting contacted before index date stratified by sex.

	Male cases (N; % [95% CI%])	Male controls (N; % [95% CI%])	Single-window regression (OR [95% CI]; p-value)	Multi-window regression (OR [95% CI]; p-value)
**Any healthcare setting**				
**Sub-total**	**245 (100%)**	**1225 (100%)**		
1 week	98; 40.0% [34.1%, 46.3%]	450; 36.7% [34.1%, 39.5%]	1.2 [1.0, 1.4]; p=0.100	0.3 [0.1, 0.6]; p=0.003
1 month	190; 77.6% [71.9%, 82.3%]	984; 80.3% [78%, 82.5%]	0.8 [0.7, 1.0]; p=0.074	0.2 [0.1, 0.5]; p=<0.001
1 year	239; 97.6% [94.8%, 98.9%]	1217; 99.4% [98.7%, 99.7%]	0.3 [0.1, 0.6]; p=<0.001	0.3 [0.1, 0.6]; p=0.003
**Primary care**				
**Sub-total**	**506 (100%)**	**2530 (100%)**		
1 week	167; 33.0% [29.1%, 37.2%]	757; 29.9% [28.2%, 31.7%]	1.2 [1.0, 1.3]; p=0.017	0.9 [0.7, 1.2]; p=0.469
1 month	346; 68.4% [64.2%, 72.3%]	1811; 71.6% [69.8%, 73.3%]	0.9 [0.8, 1.0]; p=0.012	0.7 [0.5, 0.9]; p=0.007
1 year	477; 94.3% [91.9%, 96.0%]	2410; 95.3% [94.4%, 96.0%]	0.8 [0.6, 1.1]; p=0.114	0.9 [0.7, 1.2]; p=0.453
**Emergency department**				
**Sub-total**	**330 (100%)**	**1650 (100%)**		
1 week	21; 6.4% [4.2%, 9.5%]	17; 1.0% [0.7%, 1.7%]	6.2 [3.7, 10.3]; p=<0.001	8.0 [4.7, 13.4]; p=<0.001
1 month	57; 17.3% [13.6%, 21.7%]	82; 5.0% [4%, 6.1%]	4.0 [3.1, 5.3]; p=<0.001	4.1 [3.0, 5.7]; p=<0.001
1 year	182; 55.2% [49.8%, 60.4%]	618; 37.5% [35.2%, 39.8%]	2.1 [1.8, 2.4]; p=<0.001	1.7 [1.4, 1.9]; p=<0.001
**Hospital admissions**				
**Sub-total**	**703 (100%)**	**3515 (100%)**		
Emergency admissions				
1 week	18; 2.6% [1.6%, 4.0%]	64; 1.8% [1.4%, 2.3%]	1.4 [1.0, 2.0]; p=0.034	1.6 [1.1, 2.2]; p=0.007
1 month	103; 14.7% [12.2%, 17.5%]	240; 6.8% [6.1%, 7.7%]	2.4 [2.0, 2.8]; p=<0.001	2.9 [2.4, 3.5]; p=<0.001
1 year	384; 54.6% [50.9%, 58.3%]	1638; 46.6% [45.0%, 48.3%]	1.4 [1.3, 1.5]; p=<0.001	1.2 [1.1, 1.3]; p=<0.001
Planned admissions				
1 week	<5; 0.7% [0.3%, 1.7%]	13; 0.4% [0.2%, 0.6%]	DNC	1.0 [0.5, 2.2]; p=0.995
1 month	17; 2.4% [1.5%, 3.8%]	78; 2.2% [1.8%, 2.8%]	1.1 [0.8, 1.5]; p=0.579	1.0 [0.7, 1.4]; p=0.918
1 year	114; 16.2% [13.7%, 19.1%]	652; 18.6% [17.3%, 19.9%]	0.9 [0.8, 1.0]; p=0.01	0.8 [0.7, 0.9]; p=0.004
**Hospital outpatients**				
Sub-total	579 (100%)	2895 (100%)		
Attended appointments				
1 week	23; 4.0% [2.7%, 5.9%]	159; 5.5% [4.7%, 6.4%]	0.7 [0.6, 0.9]; p=0.007	0.3 [0.2, 0.4]; p=<0.001
1 month	78; 13.5% [10.9%, 16.5%]	605; 20.9% [19.5%, 22.4%]	0.6 [0.5, 0.7]; p=<0.001	0.3 [0.2, 0.3]; p=<0.001
1 year	300; 51.8% [47.8%, 55.9%]	2294; 79.3% [77.7%, 80.7%]	0.2 [0.2, 0.3]; p=<0.001	0.3 [0.2, 0.3]; p=<0.001
Missed or cancelled appointments				
1 week	17; 2.9% [1.9%, 4.7%]	68; 2.4% [1.9%, 3.0%]	1.3 [0.9, 1.7]; p=0.168	1.5 [1, 2.1]; p=0.038
1 month	37; 6.4% [4.7%, 8.7%]	234; 8.1% [7.2%, 9.1%]	0.8 [0.7, 1.0]; p=0.015	0.8 [0.6, 1]; p=0.046
1 year	186; 32.1% [28.5%, 36.0%]	1355; 46.8% [45.0%, 48.6%]	0.5 [0.5, 0.6]; p=<0.001	0.8 [0.7, 0.9]; p=<0.001

Numbers are counts, percentages and 95% confidence intervals of individuals. Odds ratios from conditional logistic regression models for ‘any’ and each setting separately are shown. ‘Single-window regression’ models include a single dichotomous variable (1 week, 1 month or 1 year), while ‘multi-window regression’ models include a categorical variable encompassing all three time-windows in a non-overlapping way.

Supplementary Table 7. Type of health setting contacted before index date stratified by cases in NCISH and cases in SID-Cymru-only not NCISH.

	Cases in NCISH (N; % [95% CI%])	Cases in SID-Cymru not in the NCISH (N; % [95% CI%])
**Any healthcare setting**		
**Sub-total**	**255 (100%)**	**100 (100%)**
1 week	107; 42.0% [36.1%, 48.1%]	44; 44.0% [34.7%, 53.8%]
1 month	206; 80.8% [75.5%, 85.2%]	76; 76.0% [66.8%, 83.3%]
1 year	249; 97.7% [95%, 98.9%]	>95; 95.0% [88.8%, 97.9%]
**Primary care**		
**Sub-total**	**576 (100%)**	**179 (100%)**
1 week	194; 33.7% [30%, 37.6%]	75; 41.9% [34.9%, 49.2%]
1 month	404; 70.1% [66.3%, 73.7%]	127; 71.0% [63.9%, 77.1%]
1 year	544; 94.5% [92.3%, 96%]	169; 94.4% [90%, 96.9%]
**Emergency department**		
**Sub-total**	**349 (100%)**	**120 (100%)**
1 week	24; 6.9% [4.7%, 10%]	9; 7.5% [4.0%, 13.7%]
1 month	63; 18.1% [14.4%, 22.4%]	21; 17.5% [11.8%, 25.3%]
1 year	207; 59.3% [54.1%, 64.3%]	70; 58.3% [49.4%, 66.8%]
**Hospital admissions**		
**Sub-total**	**814 (100%)**	**217 (100%)**
Emergency admissions		
1 week	26; 3.2% [2.2%, 4.6%]	14; 6.5% [3.9%, 10.5%]
1 month	120; 14.8% [12.5%, 17.4%]	36; 16.6% [12.2%, 22.1%]
1 year	484; 59.5% [56.1%, 62.8%]	115; 53% [46.4%, 59.5%]
Planned admissions		
1 week	<5; 0.6% [0.3%, 1.4%]	<5; 2.3% [1.0%, 5.3%]
1 month	21; 2.6% [1.7%, 3.9%]	5; 2.3% [1.0%, 5.3%]
1 year	148; 18.2% [15.7%, 21%]	43; 19.8% [15.1%, 25.6%]
**Hospital outpatients**		
Sub-total	664 (100%)	184 (100%)
1 week	30; 4.5% [3.2%, 6.4%]	12; 6.5% [3.8%, 11.1%]
1 month	101; 15.2% [12.7%, 18.2%]	30; 16.3% [11.7%, 22.3%]
1 year	365; 55.0% [51.2%, 58.7%]	97; 52.7% [45.5%, 59.8%]
Missed or cancelled appointments		
1 week	19; 2.9% [1.8%, 4.4%]	5; 2.7% [1.2%, 6.2%]
1 month	50; 7.5% [5.8%, 9.8%]	10; 5.4% [3.0%, 9.7%]
1 year	236; 35.6% [32.0%, 39.3%]	62; 33.7% [27.3%, 40.8%]

NCISH, National Confidential Inquiry into Suicide and Safety in Mental Health; SID-Cymru, Suicide Information Database – Cymru
Numbers are counts, percentages and 95% confidence intervals of individuals.

Supplementary Table 8 (part 1/2). Accident, injury and poisoning and self-harm contacts in the year before index date stratified by sex.

	Females cases (N; % [95% CI%])	Females controls (N; % [95% CI%])	Univariate regression (OR [95% CI]; p-value)	Multivariate regression (OR [95% CI]; p-value)
**Any healthcare setting**				
**Sub-total**	**110 (100%)**	**550 (100%)**		
Accident	38; 34.6% [26.3%, 43.8%]	108; 19.6% [16.5%, 23.2%]	2.1 [1.6, 2.8]; p=<0.001	1.5 [1.1, 2.1]; p=0.024
Injury & poisoning	49; 44.6% [35.6%, 53.9%]	115; 20.9% [17.7%, 24.5%]	3.3 [2.5, 4.5]; p=<0.001	1.8 [1.2, 2.6]; p=0.004
Self-harm	36; 32.7% [24.7%, 42.0%]	55; 10.0% [7.8%, 12.8%]	4.7 [3.2, 6.8]; p=<0.001	3.5 [2.3, 5.2]; p=<0.001
**Primary care**				
**Sub-total**	**249 (100%)**	**1245 (100%)**		
Accident	14; 5.6% [3.4%, 9.2%]	53; 4.3% [3.3%, 5.5%]	1.3 [0.9, 1.9]; p=0.124	1.4 [1, 2.1]; p=0.083
Injury & poisoning	20; 8.0% [5.3%, 12.1%]	94; 7.6% [6.2%, 9.2%]	1.1 [0.8, 1.4]; p=0.661	0.8 [0.6, 1.1]; p=0.157
Self-harm	48; 19.3% [14.9%, 24.6%]	95; 7.6% [6.3%, 9.2%]	3.1 [2.4, 4.1]; p=<0.001	3.2 [2.5, 4.3]; p=<0.001
**Emergency department**				
**Sub-total**	**139 (100%)**	**695 (100%)**		
Accident	35; 25.2% [18.7%, 33%]	118; 17.0% [14.4%, 20%]	1.7 [1.3, 2.1]; p=<0.001	1.6 [1.2, 2.1]; p=0.004
Injury & poisoning	23; 16.6% [11.3%, 23.6%]	81; 11.7% [9.5%, 14.3%]	1.5 [1.1, 2.1]; p=0.01	1.2 [0.9, 1.7]; p=0.331
Self-harm	19; 13.7% [8.9%, 20.4%]	49; 7.1% [5.4%, 9.2%]	2.0 [1.4, 2.9]; p=<0.001	2.0 [1.4, 2.9]; p=<0.001
**Hospital admissions**				
**Sub-total**	**328 (100%)**	**1640 (100%)**		
Accident	34; 10.4% [7.5%, 14.1%]	87; 5.3% [4.3%, 6.5%]	2.1 [1.6, 2.8]; p=<0.001	1.2 [0.8, 1.8]; p=0.402
Injury & poisoning	120; 36.6% [31.6%, 41.9%]	229; 14.0% [12.4%, 15.7%]	3.5 [2.9, 4.2]; p=<0.001	1.6 [1.1, 2.3]; p=0.029
Self-harm	96; 29.3% [24.6%, 34.4%]	138; 8.4% [7.2%, 9.9%]	4.6 [3.7, 5.8]; p=<0.001	3.1 [2, 4.6]; p=<0.001

Numbers are counts, percentages and 95% confidence intervals of individuals. Odds ratios from univariate and multivariate (i.e., including accident, injury and poisoning, and self-harm variables) conditional logistic regression models for ‘any’ and each healthcare setting separately are shown.

Supplementary Table 8 (part 2/2). Accident, injury and poisoning and self-harm contacts in the year before index date stratified by sex.

	Male cases (N; % [95% CI%])	Male controls (N; % [95% CI%])	Univariate regression (OR [95% CI]; p-value)	Multivariate regression (OR [95% CI]; p-value)
**Any healthcare setting**				
**Sub-total**	**245 (100%)**	**1225 (100%)**		
Accident	65; 26.5% [21.4%, 32.4%]	210; 17.2% [15.1%, 19.4%]	1.8 [1.4, 2.1]; p=<0.001	1.3 [1.0, 1.6]; p=0.057
Injury & poisoning	85; 34.7% [29.0%, 40.9%]	216; 17.6% [15.6%, 19.9%]	2.6 [2.1, 3.2]; p=<0.001	1.5 [1.2, 1.9]; p=0.003
Self-harm	65; 26.5% [21.4%, 32.4%]	95; 7.8% [6.4%, 9.4%]	4.5 [3.5, 5.9]; p=<0.001	3.6 [2.7, 4.9]; p=<0.001
**Primary care**				
**Sub-total**	**506 (100%)**	**2530 (100%)**		
Accident	13; 2.6% [1.5%, 4.4%]	69; 2.7% [2.2%, 3.4%]	1.0 [0.7, 1.3]; p=0.73	0.9 [0.7, 1.3]; p=0.62
Injury & poisoning	44; 8.7% [6.6%, 11.5%]	187; 7.4% [6.4%, 8.5%]	1.2 [1, 1.5]; p=0.093	1.1 [0.9, 1.4]; p=0.29
Self-harm	72; 14.2% [11.5%, 17.6%]	145; 5.7% [4.9%, 6.7%]	3.1 [2.5, 3.9]; p=<0.001	3.1 [2.5, 3.8]; p=<0.001
**Emergency department**				
**Sub-total**	**330 (100%)**	**1650 (100%)**		
Accident	65; 19.7% [15.8%, 24.3%]	248; 15% [13.4%, 16.8%]	1.4 [1.2, 1.7]; p=<0.001	1.2 [1.0, 1.5]; p=0.075
Injury & poisoning	43; 13.0% [9.8%, 17.1%]	151; 9.2% [7.9%, 10.6%]	1.5 [1.2, 1.9]; p=<0.001	1.3 [1.0, 1.7]; p=0.08
Self-harm	57; 17.3% [13.6%, 21.7%]	84; 5.1% [4.1%, 6.3%]	3.8 [2.9, 4.9]; p=<0.001	3.7 [2.9, 4.8]; p=<0.001
**Hospital admissions**				
**Sub-total**	**703 (100%)**	**3515 (100%)**		
Accident	53; 7.5% [5.8%, 9.7%]	178; 5.1% [4.4%, 5.8%]	1.5 [1.3, 1.9]; p=<0.001	0.7 [0.5, 0.9]; p=0.004
Injury & poisoning	187; 26.6% [23.5%, 30%]	401; 11.4% [10.4%, 12.5%]	3.0 [2.6, 3.4]; p=<0.001	2.8 [2.1, 3.8]; p=<0.001
Self-harm	129; 18.4% [15.7%, 21.4%]	222; 6.3% [5.6%, 7.2%]	3.5 [3.0, 4.2]; p=<0.001	1.4 [1.0, 1.9]; p=0.032

Numbers are counts, percentages and 95% confidence intervals of individuals. Odds ratios from univariate and multivariate (i.e., including accident, injury and poisoning, and self-harm variables) conditional logistic regression models for ‘any’ and each healthcare setting separately are shown.

Supplementary Table 9. Accident, injury and poisoning and self-harm contacts in the year before index date stratified by cases in NCISH and cases in SID-Cymru-only.

	**NCISH cases (N; % [95% CI%])**	**SID-Cymru only cases (N; % [95% CI%])**
**Any healthcare setting**		
**Sub-total**	**255 (100%)**	**100 (100%)**
Accident	67; 26.3% [21.3%, 32.0%]	36; 36.0% [27.3%, 45.8%]
Injury & poisoning	93; 36.5% [30.8%, 42.5%]	41; 41.0% [31.9%, 50.8%]
Self-harm	74; 29.0% [23.8%, 34.9%]	27; 27.0% [19.3%, 36.4%]
**Primary care**		
**Sub-total**	**576 (100%)**	**179 (100%)**
Accident	21; 3.7% [2.4%, 5.5%]	6; 3.4% [1.6%, 7.1%]
Injury & poisoning	47; 8.2% [6.2%, 10.7%]	17; 9.5% [6.0%, 14.7%]
Self-harm	97; 16.9% [14.0%, 20.1%]	23; 12.9% [8.7%, 18.6%]
**Emergency department**		
**Sub-total**	**349 (100%)**	**120 (100%)**
Accident	68; 19.5% [15.7%, 24.0%]	32; 26.7% [19.6%, 35.2%]
Injury & poisoning	38; 10.9% [8.0%, 14.6%]	28; 23.3% [16.7%, 31.7%]
Self-harm	52; 14.9% [11.6%, 19.0%]	24; 20.0% [13.8%, 28.0%]
**Hospital admissions**		
**Sub-total**	**814 (100%)**	**217 (100%)**
Accident	66; 8.1% [6.4%, 10.2%]	21; 9.7% [6.4%, 14.4%]
Injury & poisoning	250; 30.7% [27.7%, 34.0%]	57; 26.3% [20.9%, 32.5%]
Self-harm	191; 23.5% [20.7%, 26.5%]	34; 15.7% [11.4%, 21.1%]

NCISH, National Confidential Inquiry into Suicide and Safety in Mental Health; SID-Cymru, Suicide Information Database – Cymru
Numbers are counts, percentages and 95% confidence intervals of individuals.
